# Identifying xenobiotic metabolites with *in silico* prediction tools and LCMS suspect screening analysis

**DOI:** 10.3389/ftox.2023.1051483

**Published:** 2023-01-18

**Authors:** Matthew Boyce, Kristin A. Favela, Jessica A. Bonzo, Alex Chao, Lucina E. Lizarraga, Laura R. Moody, Elizabeth O. Owens, Grace Patlewicz, Imran Shah, Jon R. Sobus, Russell S. Thomas, Antony J. Williams, Alice Yau, John F. Wambaugh

**Affiliations:** ^1^ Center for Computational Exposure, Office of Research and Development, U.S. Environmental Protection Agency, Research Triangle Park, Durham, NC, United States; ^2^ Southwest Research Institute, San Antonio, TX, United States; ^3^ Thermo Fisher Scientific, South San Francisco, CA, United States; ^4^ Center for Public Health and Environmental Assessment, Office of Research and Development, U.S. Environmental Protection Agency, Cincinnati, OH, United States

**Keywords:** metabolism, suspect-screening analysis, xenobiotics, *in vitro*, mass-spectrometry

## Abstract

Understanding the metabolic fate of a xenobiotic substance can help inform its potential health risks and allow for the identification of signature metabolites associated with exposure. The need to characterize metabolites of poorly studied or novel substances has shifted exposure studies towards non-targeted analysis (NTA), which often aims to profile many compounds within a sample using high-resolution liquid-chromatography mass-spectrometry (LCMS). Here we evaluate the suitability of suspect screening analysis (SSA) liquid-chromatography mass-spectrometry to inform xenobiotic chemical metabolism. Given a lack of knowledge of true metabolites for most chemicals, predictive tools were used to generate potential metabolites as suspect screening lists to guide the identification of selected xenobiotic substances and their associated metabolites. Thirty-three substances were selected to represent a diverse array of pharmaceutical, agrochemical, and industrial chemicals from Environmental Protection Agency’s ToxCast chemical library. The compounds were incubated in a metabolically-active *in vitro* assay using primary hepatocytes and the resulting supernatant and lysate fractions were analyzed with high-resolution LCMS. Metabolites were simulated for each compound structure using software and then combined to serve as the suspect screening list. The exact masses of the predicted metabolites were then used to select LCMS features for fragmentation *via* tandem mass spectrometry (MS/MS). Of the starting chemicals, 12 were measured in at least one sample in either positive or negative ion mode and a subset of these were used to develop the analysis workflow. We implemented a screening level workflow for background subtraction and the incorporation of time-varying kinetics into the identification of likely metabolites. We used haloperidol as a case study to perform an in-depth analysis, which resulted in identifying five known metabolites and five molecular features that represent potential novel metabolites, two of which were assigned discrete structures based on *in silico* predictions. This workflow was applied to five additional test chemicals, and 15 molecular features were selected as either reported metabolites, predicted metabolites, or potential metabolites without a structural assignment. This study demonstrates that in some–but not all–cases, suspect screening analysis methods provide a means to rapidly identify and characterize metabolites of xenobiotic chemicals.

## 1 Introduction

Chemical risks to public health are assessed by characterizing inherent hazards, likely exposures, and toxicokinetics (TK–that is, absorption, distribution, metabolism, and elimination by the body) (National Research Council, 1983). For many thousands of chemicals in commerce and the environment, these data are unavailable (National Academies of Sciences, Engineering, and Medicine, 2017; Isaacs et al., 2022), with TK information being scarcest (Federal Insecticide, Fungicide, and Rodenticide Act and Scientific Advisory Panel, 2014; Wetmore et al., 2015; Bell et al., 2018). Understanding chemical metabolism is necessary for risk assessment (Wang et al., 2012): Full mapping of a metabolic schema (linking parent chemicals to biologically-formed metabolites) allows better understanding of potentially toxic intermediates and ultimate products (Ioannides and Lewis, 2004; Guengerich, 2006; Tang and Lu, 2010) and identification of biomarkers of exposure (Lowry, 1995; Tan et al., 2012; Steckling et al., 2018).

New and existing chemical legislation in multiple parts of the world are drivers for faster methods of understanding key aspects of chemical behavior, including metabolism (Lilienblum et al., 2008; Wang et al., 2012; Schmidt, 2016; US Congress, 2016). Public quantitative chemical metabolism data exist for only a few thousand compounds, mostly characterizing the rate of parent chemical disappearance (Dawson et al., 2021). The lack of needed data on xenobiotic metabolism is partly due to the traditional reliance on costly time-series animal studies (Hope et al., 2008) as well as the need to develop chemical-specific analysis methods to determine concentration in experimental samples (Tolonen and Pelkonen, 2015). Though databases linking compounds with the metabolites formed in different species do exist (Spjuth et al., 2016) they tend to focus on pharmaceuticals (Piechota et al., 2013) and cover only a few hundred parent-metabolite relationships (Piechota et al., 2013; Stanfield et al., 2022).

Though traditional techniques for characterizing metabolism cannot scale to meet current needs, new approaches under development that may eventually fill data gaps: commercially available and open-source metabolism models trained on existing high-quality TK data for well-studied analytes allow rapid prediction of metabolites for any selected compound (Mekenyan et al., 2004; Marchant et al., 2008; Dimitrov et al., 2016; Djoumbou-Feunang et al., 2019). A significant drawback of these approaches is that they tend to *overpredict*, that is, predict more metabolites than actually occur (Boyce et al., 2022). Alternatively, *in vitro* studies using metabolically active cells (Shibata et al., 2002; Gomez-Lechon et al., 2008) or enzymes (Asha and Vidyavathi, 2010) can generate metabolites at lower cost than animal studies. These *in vitro* studies are limited in that they do not necessarily reproduce all *in vivo* metabolic pathways (Gouliarmou et al., 2018) and that the distribution and therefore concentration of the chemical *in vitro* may differ from what would occur *in vivo* (Gardner et al., 2022). *In vitro* metabolism studies are further limited by the requirement the development of targeted analytical chemistry methods to quantify each metabolite (Tolonen and Pelkonen, 2015). However, recently developed non-targeted analysis (NTA) methods now allow for the simultaneous identification of many chemicals in a given sample (Sobus et al., 2018). These methods can be used to enhance studies of xenobiotic metabolism *via* identification of novel metabolites (Steuer et al., 2021).

NTA methods are aided by compound screening lists, which may include existing reference spectra *or in silico* predicted spectra. These lists of chemicals anticipated to be present in a sample allow for *suspect screening analysis* (SSA). Due to the significant number of features that can be generated using NTA alone (Tolonen and Pelkonen, 2015), the process of relating those features to specific metabolites is a “significant bottleneck in deriving biological knowledge from metabolomic studies.” (Dunn et al., 2013) Trends between samples (such as increase/decrease of apparent concentration with time) may be used to reduce the number of features requiring identification (van der Hooft et al., 2017). However, even after feature reduction, there remains ambiguity in chemical identity; a single mass feature may correspond to one or more chemical formulae, and each formula may map to numerous chemical structures (McEachran et al., 2017). Tandem mass spectrometry (MS^2^) can help with eliminating some improbable structures associated with the single mass feature by focusing on features of expected compounds (for example, metabolites) (Hsieh and Korfmacher, 2009). SSA enhances NTA by focusing on only those specific features more likely to be of interest (Sobus et al., 2018). To apply SSA to a metabolism study, one might use *in silico* expert systems to reduce the search space of chemicals from hundreds of thousands of conceivable chemicals (Williams et al., 2017) to mere hundreds of plausible metabolites per compound (Boyce et al., 2022).

Recently, Kim et al. (2021) applied NTA to characterize metabolite formation for the pharmaceutical donepezil (DTXSID8048317) using samples obtained from *in vitro* metabolism experiments. Kim and colleagues observed the formation of known metabolites as well as novel metabolites characterized by m/z and retention time. The confirmed detection of a predicted metabolite, or tentative identification of a novel metabolite, can be used to: 1) better understand the metabolic pathway of a tested chemical, 2) select biomarkers of exposure to be used in future metabolic or observational studies, 3) inform follow-up dose-response studies that focus on metabolic intermediates or final products, and/or 4) identify appropriate surrogate chemicals for data poor chemicals *via* an approach often called “read-across”. In read-across, the selected surrogate chemical–also called “suitable analog” (Wang et al., 2012) and “source analogue” (Helman et al., 2019)—is used to fill data gaps for the target (data poor) chemical that is expected to behave like the selected surrogate. Metabolism is a primary principle for identifying surrogate chemicals (Wang et al., 2012). Read-across techniques are currently being used by the U.S. Environmental Protection Agency (EPA) to evaluate new chemicals under the Toxic Substances Control Act (TSCA) (Patlewicz et al., 2019) and determine Provisional Peer-Reviewed Toxicity Values (PPRTVs) for contaminants at Superfund sites (Wang et al., 2012; Lizarraga et al., 2019).

Here we evaluate the suitability of liquid-chromotography mass-spectrometry (LCMS)-based SSA methods to inform xenobiotic chemical metabolism across 33 substances. We demonstrate how *in silico* tools can be used to prepare a suspect list for SSA and guide the identification of 33 substances and their metabolites. To generate metabolites, we incubated chemicals *in vitro* with suspensions of cryopreserved primary human hepatocytes (the cells of the liver that express many of the enzymes responsible for xenobiotic metabolism) (Shibata et al., 2002). We first report on the amenability of the evaluated chemicals to SSA *via* LCMS (that is, we identify which chemicals and metabolites were suitable for detection using LCMS). We describe the generation of suspect screening lists based on the parent chemical structures and metabolite prediction software. We further demonstrate a screening level workflow for background subtraction and the incorporation of time-varying kinetics into the identification of likely metabolites. Finally, we present an in-depth analysis of haloperidol as a case study demonstrating the utility of manual curation. Computational metabolism predictions, *in vitro* metabolism assays, and SSA methods combine to provide a means to rapidly identify and characterize predicted (and even unanticipated) metabolites of chemicals that are being reviewed for safety.

## 2 Methods

**FIGURE 1:**
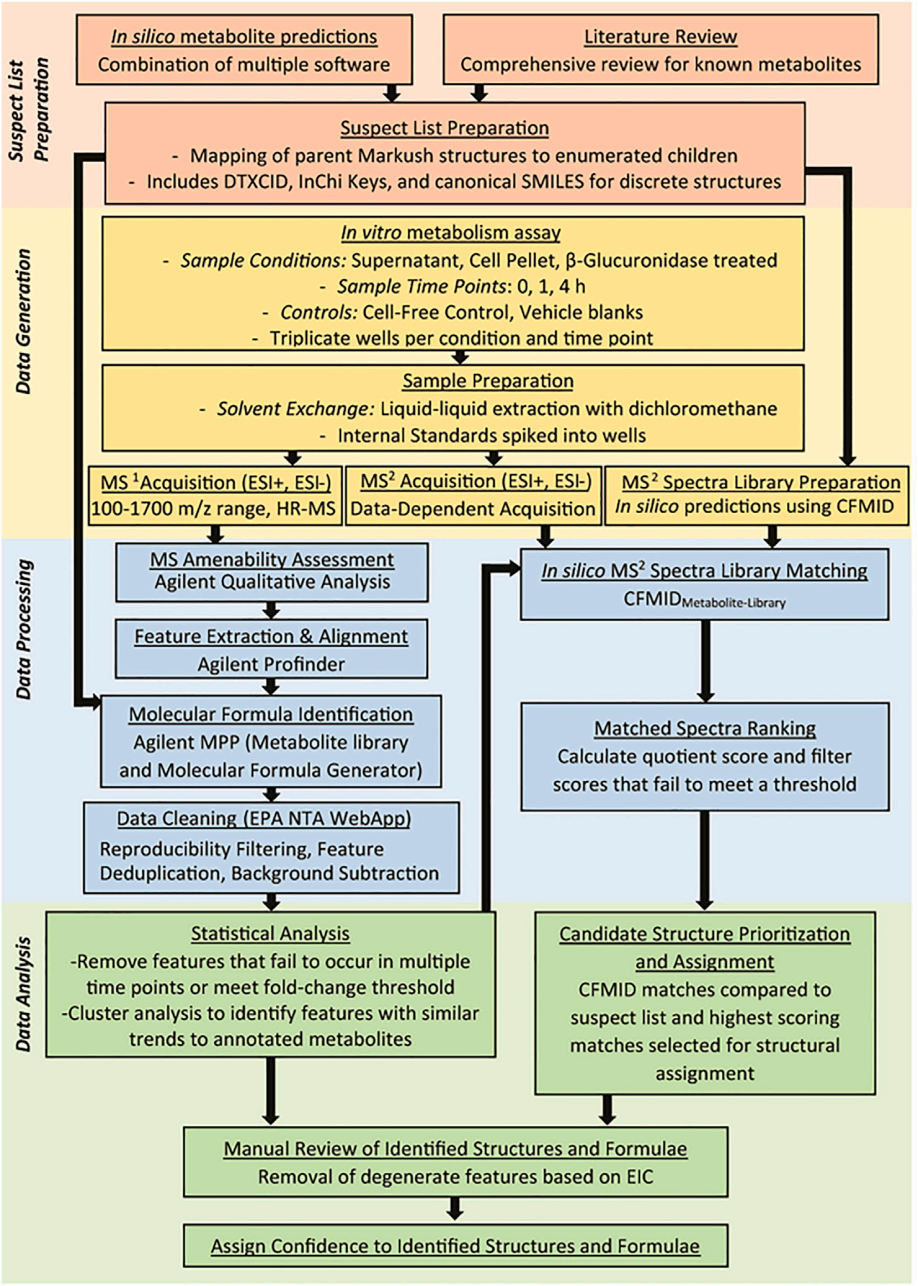
Overall workflow of the NTA method developed to identify metabolites using *in silico* tools to guide data acquisition and analysis. Each colored section represents a significant step in the analysis workflow and include generation of the suspect screening list from *in silico* tools and literature review (red section), MS^1^ and MS^2^ analysis of metabolites generated by primary hepatocytes (orange section), processing of the data to clean the data and annotate features (blue section), and data analysis to characterize features that correspond to metabolites of the parent compound (green section).

Figure 1 summarizes the workflow implemented in this study to evaluate 33 test chemicals. This workflow can be partitioned into four overarching steps: i) *Suspect List Preparation* - for each test chemical, a unique list of compounds was developed, comprised of metabolites reported in the literature and/or predicted by *in silico* models; ii) *Data Generation* - test chemicals were metabolized using an *in vitro* metabolism assay, and MS^1^ and MS^2^ data were acquired for each sample; iii) *Data Processing* - observed HRMS features were aligned, annotated, filtered, and processed to aid downstream statistical analysis and compound identification; and iv) *Data Analysis* - clusters of HRMS features were identified for selected test chemicals, and these features were flagged as potential metabolites. Observed MS^2^ spectra of flagged features were compared to *in silico* MS^2^ spectra to support metabolite identification. The sections below provide further details on each of these steps and offers insights into the strategies used for initial chemical selection.

### 2.1 Chemical selection

A total of 33 chemicals were selected for metabolic profiling. Fourteen chemicals were selected to provide data such that the methods developed here could be evaluated for utility to inform read-across. Specifically, three sets of chemical “analogs” were identified where we expected the metabolism of the mutual analogs to be similar; among each set of analogs, we expect to see similar metabolites formed. First, a pair of related compounds–methyleugenol (DTXSID5025607) and estragole (DTXSID0020575)—were selected because they are known to have similar metabolism (Rietjens et al., 2005). Methyleugenol was previously observed by Wetmore et al. (2015) to metabolize in hepatocyte cell preparations, indicating a high likelihood of success with our *in vitro* system. The next set was composed of 4-methyl-2-pentanol (DTXSID2026781) and three other analogs: 4-methyl-2-pentanone (DTXSID5021889), isopropanol (DTXSID7020762), and acetone (DTXSID8021482). The third set was composed of 3,5-dinitroaniline (DTXSID0044151) and three analogs: 2-nitroaniline (DTXSID1025726), 3-nitroaniline (DTXSID6025725), and 4-nitroaniline (DTXSID8020961).

Next, a pair of related chemicals known to produce related but distinctly different metabolites were selected—2-nitrotoluene (DTXSID4025791) and 4-nitrotoluene (DTXSID5023792) (DeBethizy and Rickert, 1984). 4-nitrotoluene has also been observed to metabolize *in vitro* in hepatocytes by Wetmore et al. (2015). We expected to observe different metabolites being formed by these two compounds despite their structural similarity.

Starting with the twelve chemicals described above and those with known metabolism data, (Dalvie et al., 2009; Wang et al., 2010; Cartus et al., 2012), chemicals were added from those chemicals in the ToxCast library for which there was either occurrence in consumer products, high exposure, or biomarker data. Twenty-one additional chemicals were selected from among the ToxCast chemical testing library (Richard et al., 2016) based upon the availability of metabolism data (Dalvie et al., 2009; Wang et al., 2010; Cartus et al., 2012), availability of exposure biomarker data for the U.S. population according to the U.S. Centers for Disease Control and Prevention National Health and Nutrition Examination Survey (NHANES) (U.S. Centers for Disease Control and Prevention, 2009), predicted high exposure rates (upper 90th percentile of predicted intake rates in Wambaugh et al. (2014)), and tentative identification in consumer products by (Phillips et al., 2018). Chemicals were added algorithmically, one-by-one to the list of test chemicals. Each addition was selected such that the fraction of the total list of high exposure, consumer product, and biomarker chemicals were roughly 90%. The tested chemicals and their physicochemical properties, as predicted with the OPERA quantitative structure-activity relationship suite (Mansouri et al., 2018) are listed in Supplementary Table S1 (S1-ChemicalsTested.xlsx). Chemical structures are provided by Supplementary Figure S1 (SFig1-ChemStructures.pptx).

### 2.2 Suspect list preparation

Preparation of the suspect screening list required sourcing metabolites from both literature and *in silico* prediction tools. The methodologies for searching literature and predicting metabolites have been previously described (Boyce et al., 2022). A summary is provided in the following sections.

#### 2.2.1 Literature Review

A list of experimentally confirmed metabolites was identified through literature review for the 33 test chemicals. Relevant publications were found by searching curated chemical databases, querying PubMed with Abstract Sifter (v4) (Baker et al., 2017), and using search engines. Metabolites recorded for this study were sourced from primary articles and include *in vitro* and *in vivo* data generated from mammalian species (specifically dog, bovine, mouse, rat, and human). In cases where multiple publications reported replicate results, the most recent publication with human metabolism data was recorded as the reference.

Metabolites identified from literature were stored in the EPA’s DSSTox database (Grulke et al., 2019). Chemicals entered into the database were assigned chemical identifiers (a substance identifier [DTXSID], as used earlier in this manuscript to identify specific substances, and the chemical structure identifier [DTXCID]) and had successor relationships mapped where a metabolite is linked to the parent compound. In those cases where experimentally identified metabolites were represented as a Markush structure, these ambiguous structures were enumerated to produce their discrete “child” structures for inclusion in the suspect screening list. An example of a Markush structure enumerated into its discrete child structures is provided in the Supplementary Material S2.

Mappings between each parent compound’s chemical identifier (DTXSID) and their respective metabolites’ identifier and associated SMILES were stored as a .CSV to serve as the suspect screening list (Supplementary Table S2: metabolite_masterlist.csv).

#### 2.2.2 *In silico* metabolite predictions

*In silico* metabolite predictions were generated for each of the 33 test chemicals using a collection of software: BioTransformer (https://biotransformer.ca) (Djoumbou-Feunang et al., 2019), Meteor (lhasalimited.org) (Marchant et al., 2008), TIMES (https://oasis-lmc.org) (Mekenyan et al., 2004), and QSAR Toolbox (https://qsartoolbox.org) (Dimitrov et al., 2016). When applicable, three generations of metabolites were predicted using Phase I and Phase II pathways. The modules and settings used for each prediction software are provided in the Supplementary Material S3.

#### 2.2.3 Suspect list compilation

The results from the literature review and metabolite prediction software were merged into a single dataset. A total of 2062 metabolites were included in the suspect list, with 1723 unique metabolites (Supplementary file: metabolite_masterlist.csv). The structure is encoded as a SMILES (‘SMILES’ column) with additional descriptors: monoisotopic mass (‘MW’ column), molecular formula (‘Formula’ column). Unique identifiers for the metabolite (“Metabolite_DTXSID” column) and parent compound (“Parent_DTXSID” column) are also provided, as is a Boolean column to indicate whether the metabolite is part of a Markush structure (“Markush” column). Metabolites generated by prediction software were included as part of the MS^2^ Data-Dependent Acquisition (DDA) preferred ions list (as indicated by the “DDA_Included” column). Finally, the source of the metabolite (“BioTransformer”, “Meteor”, “Toolbox”, “TIMES”, and “Reported” columns) was added. InChI Keys were used to ensure no structures were duplicated in the list (the keys are not included in the provided table).

For the purpose of assigning a molecular formula during the MS^1^ analyses, the suspect screening list was reduced to 538 unique molecular formulae that had a molecular weight greater than 100 Da.

### 2.3 Data generation

#### 2.3.1 *In vitro* metabolism assay

Metabolites were generated by incubating test chemicals *in vitro* with pooled, cryopreserved human hepatocytes (Shibata et al., 2002; Gómez-Lechón et al., 2008). This assay has been used to characterize the rate of parent chemical metabolism for more than a thousand chemicals in commerce (Breen et al., 2021). Pooled human cryopreserved hepatocytes (Thermo Fisher, HMCS10) were prepared at a concentration of 1 × 10^6^ cells/mL using William’s E Medium (Gibco, Cat#: A1217601) supplemented with Maintenance Supplement Kit (Gibco, Cat#: CM400). Dosage solutions for each of the selected chemicals outlined in Section 2.1 were prepared at 20 μM by diluting 20 mM stock solutions of the respective chemical in dimethyl sulfoxide (DMSO) (Thermo Fisher, 043998.M1) with cell media. Equal volumes of the cells suspension and dosage solutions were aliquoted into 96-well plates (Greiner Bio-One, Cat#: 650261) and incubated on an orbital shaker at 5% CO_2_ and 37°C for 0, 1, or 4 h. After the incubation period, the plates were sealed and transferred to a −80°C freezer to terminate the reaction and lyse the cells. Each time point was prepared in triplicate across three separate plates. Additionally control plates were incorporated into the assay: a DMSO vehicle blank, where the dosage solution contained only DMSO; and cell-free controls, where no cells were added to the well.

After at least 18 h at −80°C, the plates were thawed and centrifuged. Supernatant was removed from each well and partitioned into two equal aliquots. One aliquot was returned as is to −80°C. The other aliquot was deconjugated by treating with an equal volume of reaction mixture containing purified >5 U/mL β-glucuronidase buffered to pH ∼5 (Abalonase^®^+, Ango Life Sciences). The treated samples were incubated at 45°C–65°C for at least 2 h, then the reaction was terminated by adding an equal volume of acetonitrile (ACN) (Thermo Fisher, 043166.AK). A summary of the metabolism assay and the total samples generated from this workflow are outlined in the Supplementary Material S1.

#### 2.3.2 Sample preparation

Each well of the 96-well plates used as part of the *in vitro* metabolism assay were treated with 100 μL of dichloromethane (DCM) containing several internal standards: 1,4-dichlorobenzene-D_4_ (DTXSID30959416), naphthalene-D_8_ (DTXSID10894058), acenaphthene-D_10_ (DTXSID40893473), phenanthrene-D_10_ (DTXSID60893475), chrysene-D_12_ (DTXSID00893474) and perylene-D_12_ (DTXSID60934397). Samples were then manually transferred to 2.0 mL autosampler vials, where the DCM layer was removed for use in a GC-MS analysis, which is not covered in this publication. The samples were then diluted with water by a factor of three and spiked with 100 μL of methanol containing 3-phenoxybenzoic acid-^13^C_6_ (DTXSID101028020) and diisopropyl methylphosphonate-D_14_ (DTXSID201348522), which were used as internal standards for negative and positive ion modes, respectively. Each standard used for LCMS analysis was added at a concentration of 100 ppb.

#### 2.3.3 Acquiring LCMS^1^ and MS^2^ data

All samples and controls were analyzed *via* LCMS using an Agilent 1,290 Infinity high performance liquid chromatograph (HPLC) coupled to an Agilent 6,540 Ultra High Definition (UHD) quadrupole time-of-flight (Q-TOF) mass spectrometer. A detailed outline of the experimental conditions, instrument parameters, and injection order are provided in the Supplementary Material S3. Each set of triplicate preparations was measured using full scan mode (MS^1^) with a single injection (20 μL) for each ionization mode. The final replicate for each triplicate set was injected an additional time (with a DDA method to collect MS^2^ spectra, where charged monoisotopic masses ([M + H] for positive ion mode and [M-H] for negative ion mode). DDA was performed independently of the suspect screening list; after review of the MS-level data, potential detections from the suspect screen included as a preferred-ions list that were not selected for fragmentation *via* DDA were targeted for fragmentation in an additional injection.

#### 2.3.4 MS^2^ spectra library preparation

*In silico* MS^2^ spectra were generated for each structure stored in the suspect screening list using the freely available CFM-ID 2.0 algorithm (Allen et al., 2015). The SMILES strings in the suspect list were used as the input for the CFM-ID algorithm (https://sourceforge.net/projects/cfm-id), and predictions were generated using the settings for electrospray ionization, both positive and negative ion mode, using three collision energies (10, 20, and 40 eV). Predicted spectra were linked to structure metadata (for example, DTXCID, molecular formula, and monoisotopic mass) and stored as a database (hereafter referred to as the CFM-ID database) (McEachran et al., 2019).

### 2.4 Data processing

The MS^1^ data were processed using a series of steps with increasing scrutiny to filter the data into relevant features. The initial pass of the MS^1^ data was used to determine whether compounds within the suspect screening list were present in a parent chemical’s dataset. Parent chemicals that were both amenable to LCMS and hepatocyte metabolism were selected for subsequent analysis, where more precise feature extraction was performed, and custom chemical libraries were used to annotate those features. Data cleaning steps were used to further reduce the list of features and calculate relevant statistics. Finally, MS^2^ spectral libraries were used to support the annotation of select features. Data are summarized in Supplementary Table S3: S3-Hepatocyte_Results_LC_Parents_and_Metabolites.xlsx.

As part of data processing, the quality of the MS^1^ were evaluated by tracking the retention time and mass error of each internal standard. A description of how the quality measurements were recorded and a summary of the quality data are provided in the Supplementary Material S8.

#### 2.4.1 Identification of amenable chemicals

Agilent’s Qualitative Analysis software (v. 10.0) was used to automate the extraction and integration of feature peaks in the MS^1^ data as part of the initial evaluation to identify parent chemicals amenable to the experiment. Features were selected using a 50 ppm mass window and either [M + H]^+^ ions for positive-ion mode or [M-H]^-^ ions for negative-ion mode. Extracted ion chromatograms (EICs) were manually evaluated and features present across multiple samples of a single test chemical but absent in the DMSO vehicle and other samples were flagged as potential metabolites. Parent chemicals that were not detected by LCMS or failed to have potential metabolites were excluded from subsequent analysis.

Parent chemicals undergoing transformations were identified by calculating the relative change in signal between the 0 h and 4 h time points. For these calculations, the signal for the 0 h and 4 h time points is the average peak area of triplicate wells. A subset of six test chemicals were selected to develop an analysis workflow for the identification of metabolites. These chemicals were selected using three criteria: 1) the parent chemical was detected by LCMS analysis, 2) potential metabolites were present within the dataset, and 3) a range of metabolic responses were represented by these test chemicals (that is, these chemicals range between high to low relative changes over the course of the experiment). The final list of selected chemicals was: celecoxib (DTXSID0022777), CP-122721 (DTXSID9047251), curcumin (DTXSID8031077), dapsone (DTXSID4020371), haloperidol (DTXSID4034150), and sulindac (DTXSID4023624).

#### 2.4.2 Feature extraction and alignment

Agilent’s MassHunter Profinder software (v. B.08.00) was used to align and recursively extract molecular features from MS1 data. Each of the six selected test chemicals were processed in batches, where a single batch included all time points (for example, 0, 1, and 4 h) and conditions (for example, supernatant, cell pellet, and β-glucuronidase treated) for the test chemical, as well as the DMSO vehicles measured across each condition. Triplicate preparations were grouped together as part of the analysis, and molecular features were extracted if found in two of the three replicates. Settings for the analysis are provided in Supplementary Material S4. Lists of extracted features were stored as.CEF files and used for subsequent analysis.

#### 2.4.3 Molecular formula identification

Extracted features were processed by Agilent’s Mass Profiler Professional (MPP, v. 15.1), which assigned molecular formula using two Personal Compound Database Libraries (PCDL) applied successively (identification settings provided in Supplementary Material S5). The first PCDL was a subset of unique molecular formulae pulled from the suspect screening list associated with the parent chemical being processed. The second PCDL was a list of unique formulae identified in human blood plasma derived from EPA’s CompTox Chemicals dashboard (https://comptox.epa.gov/dashboard/chemical-lists/HUMANBLOOD) (Williams et al., 2017). Features identified by the human blood plasma PCDL were considered endogenous to the sample and not a transformation product of the dosed test chemicals, therefore, these features were excluded from subsequent analysis. Features not annotated by PCDLs were assigned theoretical formulas using Agilent’s molecular formula generation algorithm (Supplementary Material S5). Lists of annotated features and their respective abundance values were exported as ‘peak list’ data in a.csv format.

#### 2.4.4 Data cleaning

Peak list data were processed using adjusted source code from the EPA’s NTA WebApp (https://github.com/quanted/nta_app). The adjusted source code is hosted on GitHub (https://github.com/USEPA/CompTox-ExpoCast-SSAMetabolism) and performed the following data cleaning steps: 1) separate data into individual conditions and time point pairings, 2) remove duplicate features, 3) calculate summary statistics, 4) remove irreproducible features, 5) flag adducts, and 6) combine results into summary files. An expanded explanation of the script and data cleaning steps are provided in the Supplementary Material S6.

#### 2.4.5 *In silico* MS^2^ spectra library matching

Positive and negative ion mode MS^2^ data collected as.d files were converted into mascot generic files (.mgf) to facilitate *in silico* library matching. Features extracted from the MS^2^ data were compared to the features-of-interest derived from the MS^1^ analysis workflow, and the subset of overlapping features between the datasets were queried against the CFM-ID database. Comparisons between monoisotopic masses were based on an error window of 10 ppm. Each candidate structure returned from the database included CFM-ID spectra predicted at 10, 20, and 40 eV collision energies (CE). Similarity scores were calculated between the experimental MS^2^ spectra and each collision energy using a composite dot product (Stein and Scott, 1994), and the resulting scores were summed into a single aggregate value (Chao et al., 2020). MS^2^ feature filtering, database querying, and similarity calculations were handled using python scripts hosted on a GitHub repository (https://github.com/USEPA/CompTox-ExpoCast-SSAMetabolism); a summary of these scripts are provided in the Supplementary Material S7.

#### 2.4.6 Matched spectra Ranking

Candidate structures for each MS^2^ feature were ranked using quotient scores, which were calculated by normalizing all similarity scores of a candidate feature to the highest similarity score assigned to that feature (Chao et al., 2020). Quotient scores range from 0 to 1 and provide a relative comparison across all candidate compounds (having *in silico* MS^2^ spectra) with a precursor mass matching that of a MS^1^ feature of interest. Matches with a quotient score <.75 were removed from the data prior to candidate prioritization.

### 2.5 Data analysis

#### 2.5.1 Statistical analysis

Custom Jupyter notebooks were used to perform additional data processing steps (for example, data imputation and standardization) and cluster analysis to select a subset of features suspected to be metabolites of the test chemical. These notebooks are hosted on a public GitHub repository (https://github.com/USEPA/CompTox-ExpoCast-SSAMetabolism). Each script performed a series of steps: 1) remove all features flagged as potential adducts, 2) remove features that failed to show a fold-change increase beyond a set threshold, 3) cluster similarly behaving features, and 4) export a list of features suspected to be metabolites of the parent chemical.

Adduct removal was performed by eliminating all features flagged with a ‘1’ value in the ‘Is_Adduct_or_Loss’ column. Fold-change calculations were performed on the reduced feature list to determine the relative change in abundance between the 0 h timepoint and later time points (1 h and 4 h) for each test condition. Data imputations were performed if no abundance values were measured at a time point: if no values were measured for the 0 h time point, the maximum measured fold-change for that condition was imputed for the 1 or 4 h time point. If no abundance was measured for the 1 h or 4 h time point, the minimum measured fold-change for that condition was imputed at that time point. Features were removed from the data if they were not measured in at least two replicates of a condition and failed to have a fold-change ≥1.5 for one of those replicates.

After these initial filtering steps, the remaining features were analyzed using the median background-subtracted values calculated as part of the data cleaning step (Section 2.4.4). Features that were not detected at individual time points were imputed with the peak height threshold used for feature extraction (10,000). The background-subtracted values were log_2_-transformed then z-normalized. Similarly behaving features were clustered using a K-means clustering algorithm available in the scikit-learn Python package (Pedregosa et al., 2011). The number of clusters (k) were optimized for each dosed chemical using the inertia metric (elbow method) and ranged from 15—20. Clusters that included one or more features annotated as a metabolite from the suspect screening list were pooled into a list of features-of-interest. These features represent a combination of candidate metabolites and unidentified features that exhibited similar behavior across the sample conditions and time points.

#### 2.5.2 Candidate structure prioritization and assignment

Custom Jupyter notebooks were used to compare structural assignments between the CFM-ID spectra match and metabolites in the suspect screening list. Intersecting structures were identified by looking up InChI keys between the two lists, and the features were assumed to be a metabolite if a match was found. If no structures were assigned to a feature of interest through spectra matching, the MS^1^ level data (for example, accurate monoisotopic mass) were compared against the suspect screening list.

#### 2.5.3 Manual review of identified structures and formulae

EICs of each feature of interest were prepared using Agilent’s Qualitative Analysis software. These chromatograms were manually reviewed to ensure no degenerate features were included as part of the reported potential metabolites.

#### 2.5.4 Assign confidence of identified structures and formulae

Recalling that we only matched features observed to demonstrate intensity changes over time as incubated with hepatocytes, metabolite identification was assigned using three levels of confidence derived from Schymanski et al. (2014): level 2b, a probable structure is assigned based on favorable matching between the experimental MS^2^ spectrum and predicted CFM-ID spectra; level 3, a candidate structure is assigned based on matches between the structures on the suspect screening list (that is known or possible metabolites) and the mass pulled from MS^1^ data; and level 5, a feature’s exact mass is known and a candidate molecular formula was assigned using the molecular formula generator. 2a was not possible because no metabolites were present in the reference libraries (i.e., Agilent PCDLs). We note that our level 3 designations might reasonably be level 4, since that is (by definition) a match at the formula level; the experimental evidence supporting a level 3 distinction here depends on the observation of a time-changing signal for a formula corresponding to a predicted metabolite in the media where a known parent chemical was metabolized.

## 3 Results

### 3.1 Preparation of a suspect screening list using *in silico* tools and literature

The suspect screening list was prepared by combining metabolites originating from two sources: theoretical metabolites predicted by *in silico* tools, and experimentally confirmed metabolites reported in the literature. Table 1 summarizes the metabolite counts for each parent compound used in this study and the sources from which those metabolites were extracted. The metabolites used for the suspect screening list are provided as a supplementary file (Supplementary Table S2: S2-metabolite_masterlist.csv), which maps chemical descriptors for each metabolite (that is, SMILES, formula, monoisotopic mass) to their parent compound’s substance identifier (DTXSID). Additional metadata are included for each metabolite: the source of the metabolite (literature and/or prediction tool), whether the metabolite is represented as a Markush structure in literature, and whether the metabolite was included as part of the MS^2^ DDA preferred-ions list.

**TABLE 1:** Summary of reported and predicted metabolites for each parent compound.

Parent compound	DTXSID	#Reported metabolites	Literature sources	#Predicted metabolites^a^			
				BT	M	T	TB
o-Aminoazotoluene	DTXSID1020069	7	Samejima et al. (1967)	60	12	1	8
2-Nitroaniline	DTXSID1025726	8	Nystrom and Rickert (1987)	14	4	0	5
2-Nitrotoluene	DTXSID4025791	0	-	14	7	0	11
Isopropanol	DTXSID7020762	2	Slaughter et al. (2014), Nordmann et al. (1973)	0	11	1	7
3,5-Dinitroaniline	DTXSID0044151	0	-	9	4	0	4
3-Nitroaniline	DTXSID6025725	7	Nystrom and Rickert (1987)	18	7	0	5
Estragole	DTXSID0020575	8	Punt et al. (2009)	11	16	10	11
4-Methyl-2-pentanol	DTXSID2026781	3	McGinty et al. (2010), McGinty et al. (2010), Gingell et al. (2003)	2	7	3	5
4-Methyl-2-pentanone	DTXSID5021889	2	McGinty et al. (2010), Gingell et al. (2003)	0	5	2	6
4-Nitroaniline	DTXSID8020961	6	Nystrom and Rickert (1987)	14	7	0	3
4-Nitrotoluene	DTXSID5023792	4	DeBethizy and Rickert (1984)	11	8	0	10
Acetone	DTXSID8021482	4	Casazza et al. (1984)	0	32	1	7
Acrylamide	DTXSID5020027	10	Fennell et al. (2005), Fuhr et al. (2006)	1	6	1	3
BDE-209	DTXSID9020376	3	Macholz et al. (1982), Huwe and Smith (2007)	0	382	1	6
Benzoic acid	DTXSID6020143	2	Abdo et al. (1985), Chidgey and Caldwell (1986), Chidgey et al. (1986)	13	2	1	0
Benzyl acetate	DTXSID0020151	9	Abdo et al. (1985), Chidgey and Caldwell (1986), Chidgey et al. (1986)	15	5	4	4
Benzyl alcohol	DTXSID5020152	4	Abdo et al. (1985), Chidgey and Caldwell (1986), Chidgey et al. (1986)	22	4	5	2
Benzyl butyl phthalate	DTXSID3020205	11	Chidgey et al. (1986), Nativelle et al. (1999)	82	21	10	15
beta-Hexachlorocyclohexane	DTXSID7020685	10	Feil et al. (1973)	2	2	1	8
Bisphenol A	DTXSID7020182	4	Pritchett et al. (2002)	8	2	2	2
Butylated hydroxytoluene	DTXSID2020216	0	-	21	9	15	4
Celecoxib	DTXSID0022777	4	Paulson et al. (2000)	18	3	8	11
CP-122721	DTXSID9047251	13	Kamel et al. (2006), Colizza et al. (2007)	84	23	7	12
Curcumin	DTXSID8031077	13	Ireson et al. (2001), Pan et al. (1999),Prasad et al. (2014)	20	17	6	11
Dapsone	DTXSID4020371	8	Zuidema et al. (1986)	16	6	5	1
Dieldrin	DTXSID9020453	6	Hutson (1976)	3	1	3	1
Haloperidol	DTXSID4034150	7	Johansson et al. (2010)	132	19	7	8
Lindane	DTXSID2020686	0	-	6	2	7	10
Methyleugenol	DTXSID5025607	12	Cartus et al. (2012)	15	26	10	15
Naphthalene	DTXSID8020913	11	Ayala et al. (2015)	25	10	4	7
o,p'-DDT	DTXSID6022345	14	Feil et al. (1973)	13	2	1	0
Sulindac	DTXSID4023624	6	Swanson and Boppana (1981)	50	8	5	20
Zileuton	DTXSID9023752	2	Machinist et al. (1995), Sweeny and Nellans (1995)	14	2	3	5

^a^ Metabolite prediction software are abbreviated as follows:

BT, BioTransformer; M, meteor; T, times; TB, toolbox.

#### 3.1.1 Generation of metabolites using *in silico* tools

**FIGURE 2:**
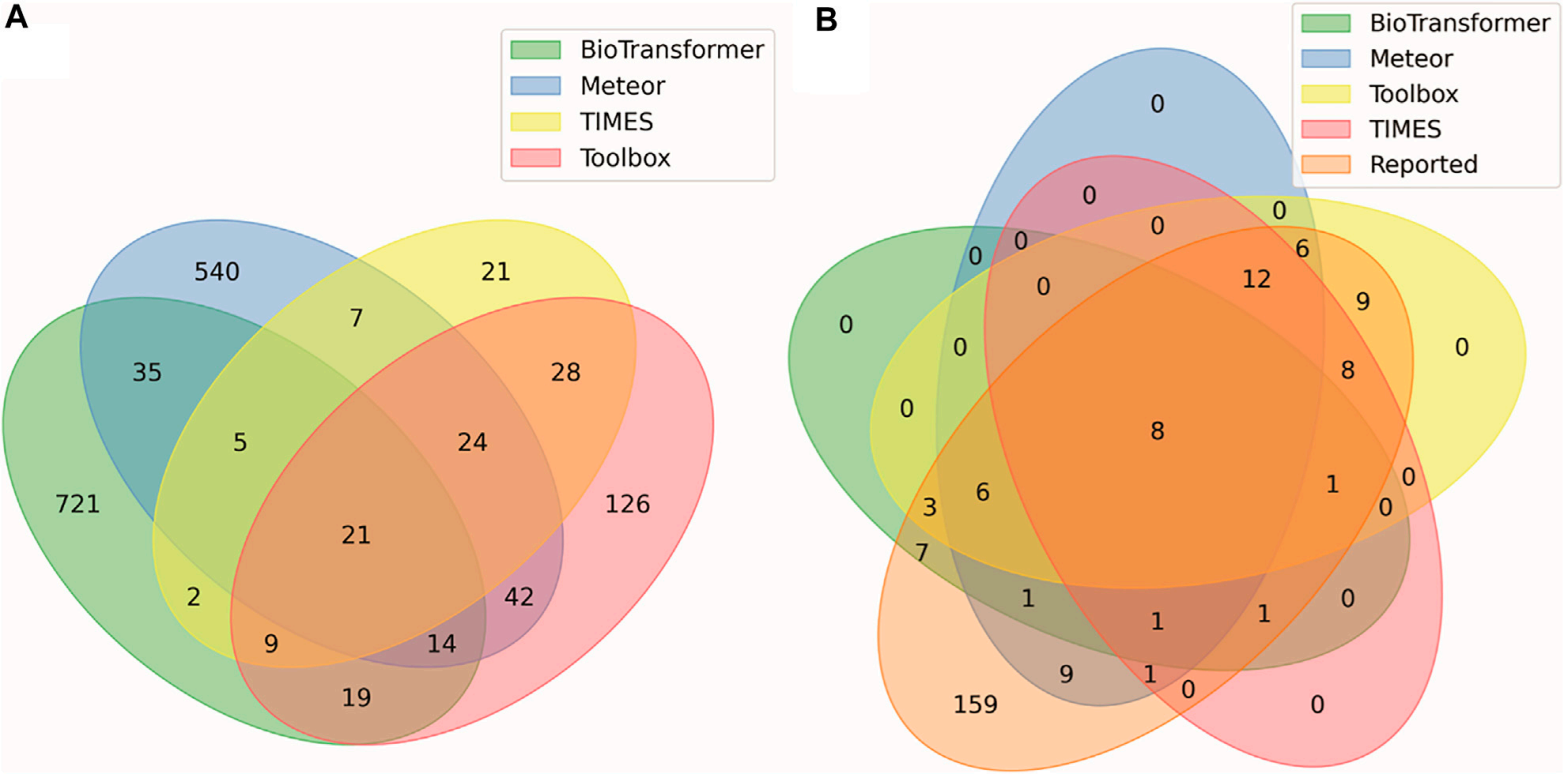
Venn diagram displaying concordance between **(A)** all predicted metabolites and the *in silico* tools that generated the prediction, and **(B)** all metabolites reported in literature and *in silico* tools that generated those predictions. Numbers indicate how many metabolites match the conditions of each ellipse. For example, among the predicted metabolites in **(A)** there were 28 metabolites predicted by both TIMES (https://oasis-lmc.org) (Mekenyan et al., 2004), and QSAR Toolbox (https://qsartoolbox.org) (Dimitrov et al., 2016) but not by the other prediction methods. Among the metabolites reported in the literature in **(B)**, only 8 of the 28 predicted by only TIMES and ToolBox were previously reported.

Table 1 summarizes the number of predictions generated for each parent compound using four *in silico* tools: BioTransformer, Meteor, TIMES, and QSAR Toolbox. These models simulated reaction pathways including Phase I metabolism (oxidative, reduction, and hydrolysis) and Phase II transformations (including glucuronidation, glutathione conjugation, sulfation, and N-acetylation). The QSAR Toolbox and TIMES models include over 600 biotic and abiotic reactions commonly seen in xenobiotic metabolism. BioTransformer predicted the greatest number of metabolites (827), followed by Meteor (714), the Toolbox (316), then TIMES (132). Of the 1989 total predicted metabolites, 1,668 of these structures were unique with 581 predictions (∼30% of total predictions) overlapping between the different models. Figure 2 shows the number of overlapping predictions between each software application. The information presented in Figure 2 can be used to assess similarity: 721 (87%) of BioTransformer’s predictions were not replicated by other software, whereas Meteor, Toolbox, and TIMES predicted 540 (76%), 126 (40%), and 21 (7%) unique metabolites, respectively. BioTransformer and Meteor were the most dissimilar models, with the majority of predictions being unique to their respective models. TIMES and the Toolbox showed the greatest overlap, with 62% of TIMES’s predicted metabolites (82 in total) overlapping with those of Toolbox. The similarity between these software applications is unsurprising, as they share a common knowledge base developed by the Laboratory of Mathematical Chemistry (LMC, University “Prof. As. Zlatarov”, Bourgas, Bulgaria). Comparisons between the different models used in this study are in concordance with previous efforts comparing the performance of *in silico* metabolite predictions tools (Boyce et al., 2022). Using several prediction models to prepare the suspect screening list expands the chemical space being monitored for each test chemical. By prioritizing chemical coverage, we limit the possibility of missing identifications from the *in vitro* analysis. As such, all predicted metabolites were included in the suspect screening list prior to the MS^2^ DDA.

#### 3.1.2 Curating metabolites reported in literature

Table 1 summarizes the distribution of metabolites identified in the literature for each of the dosed compounds. A total of 234 metabolites were extracted from literature, with 225 represented as discrete structures and the remaining 9 reported as Markush representations. These Markush structures were enumerated and mapped to 43 discrete child structures prior to incorporation into the suspect screening list. Unique identifiers (DTXSIDs) were generated for each reported metabolite and the Markush children were given the same DTXSID to maintain their relationship to a common parent structure. Metabolites identified by *in vitro* and *in vivo* human metabolism studies were always prioritized for inclusion into the suspect screening list; however, metabolites identified by *in vitro* or *in vivo* studies using rats, mice, or dogs were included if no human data were available. Of the 33 parent compounds investigated in this study, no reported metabolites were included for 2-nitrotoluene, 3,5-dinitroaniline, butylated hydroxytoluene, or lindane. While there are studies that identify metabolites for lindane (Fitzloff et al., 1982), 2-nitrotoluene (DeBethizy and Rickert, 1984) and butylated hydroxytoluene (Conning and Phillips, 1986), these articles were not identified by the authors prior to generation of the MS^2^ spectra suspect screening library.

#### 3.1.3 Combining metabolite data and summary of the suspect screening list

Figure 2 summarizes the overlap between metabolites reported in literature with those predicted by software, in which 75 (32%) of reported metabolites were predicted by some combination of *in silico* tools. Conversely, these data show that there were 159 metabolites previously observed in the literature, not counting individual Markush children, that were not predicted *in silico*. The literature metabolites were added to the suspect screening list to augment the *in silico* predictions. As previously noted in Section 2.2.3, metabolites reported in literature were not included as part of the DDA preferred-ions list; therefore, these 159 metabolites were only available for identification in MS^1^ data.

### 3.2 Identifying parent compounds undergoing *in vitro* metabolization by human hepatocytes

MS^1^ data collected for each of the starting chemicals were analyzed to examine the extent of metabolization over the course of the 4-h study. Representative spectra for each chemical are provided *via* GitHub at https://github.com/USEPA/CompTox-ExpoCast-SSAMetabolism/blob/main/Data/Hepatocyte_Mass_Spectra_LC.pptx. Of the starting chemicals, only 12 were measured in at least one sample in either positive or negative ion mode. The relatively low number of measured parent ions is unsurprising, as these chemicals were selected to represent a broad range of physicochemical properties and cover regions not commonly amenable to LC-ESI-MS. These properties include relatively high vapor pressure (for example, acetone, isopropanol, 4-methyl-2-pentone) or a lack of strong intramolecular dipole (for example, BDE-209), which are both correlated to poor electrospray ionization efficiency (Kiontke et al., 2016). GCMS analysis was used as an orthogonal method to analyze features not amenable to LCMS, and the full analysis of these data is pending.

**FIGURE 3:**
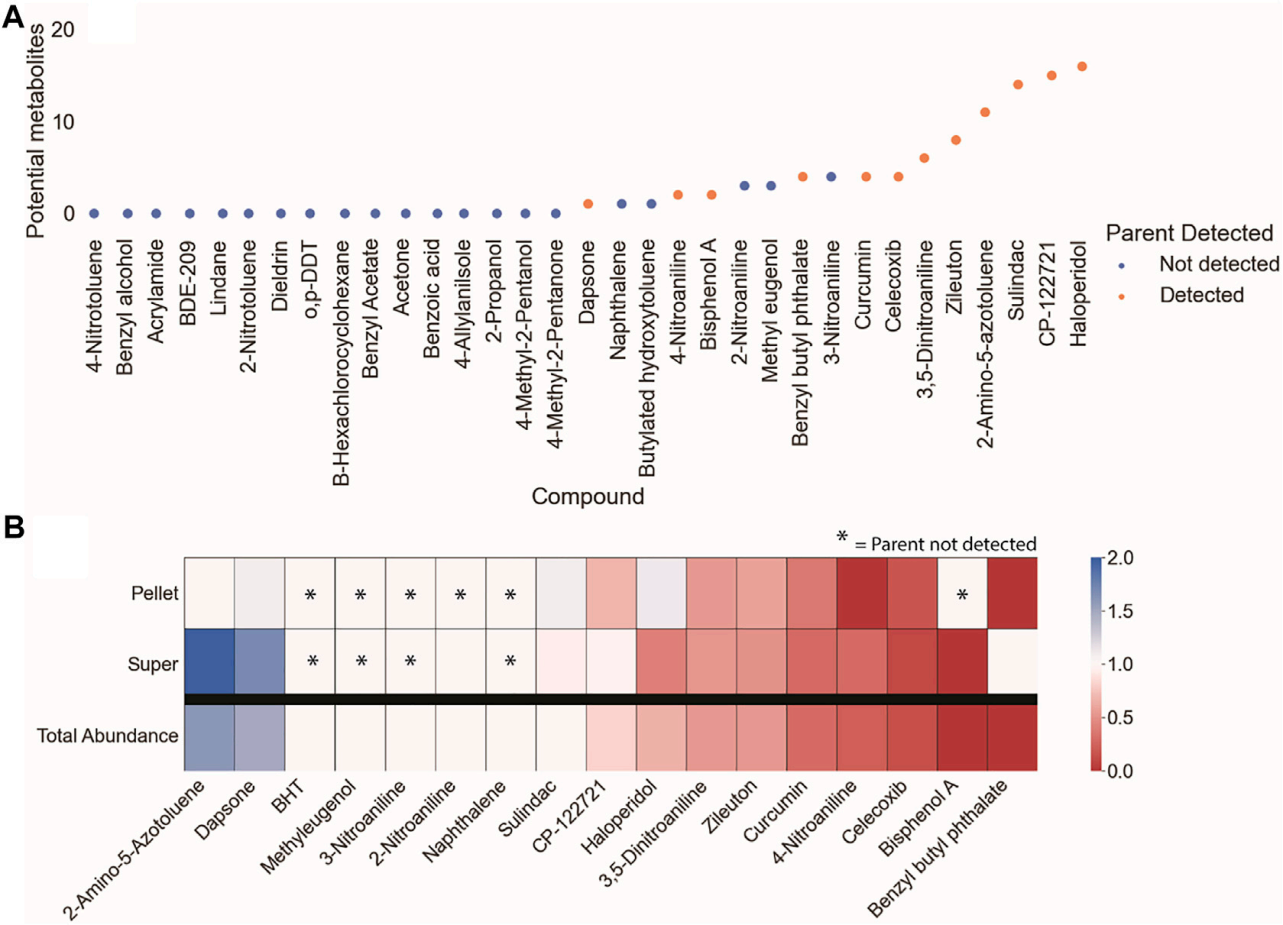
Preliminary survey of MS^1^ data to prioritize data processing and analysis of parent compounds. EIC of the parent compounds were used to assess **(A)** whether the parent compound was detectable and the quantity of potential metabolites within that dataset, and **(B)** the fold-change of the parent compound over the course of the experiment. Parent compounds that were detectable, had relatively high potential metabolite counts, and decreased over the course of the experiment were prioritized for metabolite identification.

Potential metabolites were identified for 17 of the starting compounds, which included all 12 of the parent compounds that were measurable by LCMS. The increase in the number of samples with possible metabolites (17) relative to measurable parents (12) is expected, as the metabolism of xenobiotic substances often increases the polarity of the substance which improves ionization efficiency for MS detection. The subset of parent chemicals with potential metabolites are outlined in Figure 3A.

The depletion of the parent chemicals over the course of the *in vitro* assay was evaluated by comparing the fold-change difference in peak area between the start and end of the experiment (Figure 3B). Fold-change calculations for the cell pellet and supernatant involve both the metabolism and partitioning of the parent chemical between the two conditions. The total abundance (sum of the peak intensities for the cell pellet and supernatant samples) was calculated to provide a generalized readout of overall metabolism without the need to account for partitioning. The parent chemicals that were both measurable by LCMS analysis and had potential metabolites were ranked by their degree of depletion, from least to most depleted: 2-amino-5-azotoluene (DTXSID1020069), dapsone (DTXSID4020371), sulindac (DTXSID4023624), CP-122721 (DTXSID9047251), zileuton (DTXSID9023752), haloperidol (DTXSID4034150), 3,5-dinitroaniline (DTXSID40210803), bisphenol A (DTXSID7020182), benzyl butyl phthalate (DTXSID3020205), curcumin (DTXSID8031077), and celecoxib (DTXSID0022777) (Figure 3B). Of these compounds, dapsone and 2-amino-5-azotoluene exhibited total abundance values >1 (1.05 and 1.75, respectively). The increase in signal of 2-amino-5-azotoluene is accompanied by high relative standard deviations for intra-condition measurements, exceeding 110% for the 0-h cell pellet sample. Manual inspection of these data confirmed the presence of aberrant peaks in at least one of the replicates of the 0-h samples; however, peak shapes improved at later time points and were the cause for the increase in relative abundance over the course of the experiment.

### 3.3 Developing an analysis workflow to identify metabolites: Haloperidol as a case study

Metabolite identification was performed by: 1) processing the MS^1^ data to extract and annotate molecular features, 2) filtering the data to remove features that are unlikely to be metabolites of parent compounds, 3) analyzing remaining features to identify clusters that correspond to metabolites (known or predicted) of the parent compounds; and 4) comparing MS^2^ spectra of the clustered features against an *in silico* MS^2^ library to assign probable structures. Many decisions had to be made throughout the analysis workflow. For example, the parameters for feature selection and annotation, the criteria for removal of irrelevant features, and choice of k-means cluster optimization. Due to this complexity, a candidate compound was needed to develop the analysis methodology before application to other dosed compounds. Haloperidol was selected as the candidate compound due to its highest observed number of potential metabolites in the initial survey of MS^1^ data (Figure 3A). Haloperidol also exhibits a moderate degree of metabolism (∼35% decrease of parent compound) over the 4-h incubation (Figure 3B).

#### 3.3.1 Feature extraction and annotation

Recursive feature extraction of the samples treated with haloperidol MS^1^ data found 697 features in positive ion mode and 1,164 features in negative ion mode. Eleven features were annotated as potential matches to structures in the suspect screening list—seven in positive mode and four in negative mode. Of these annotations, C_21_H_23_ClFNO_2_ (haloperidol) was reported in positive mode and only one feature—C_27_H_31_ClFNO_8_ (glucuronidated haloperidol)—was reported in both ionization modes. The remaining eight features had unique molecular formulae assigned to them.

**FIGURE 4:**
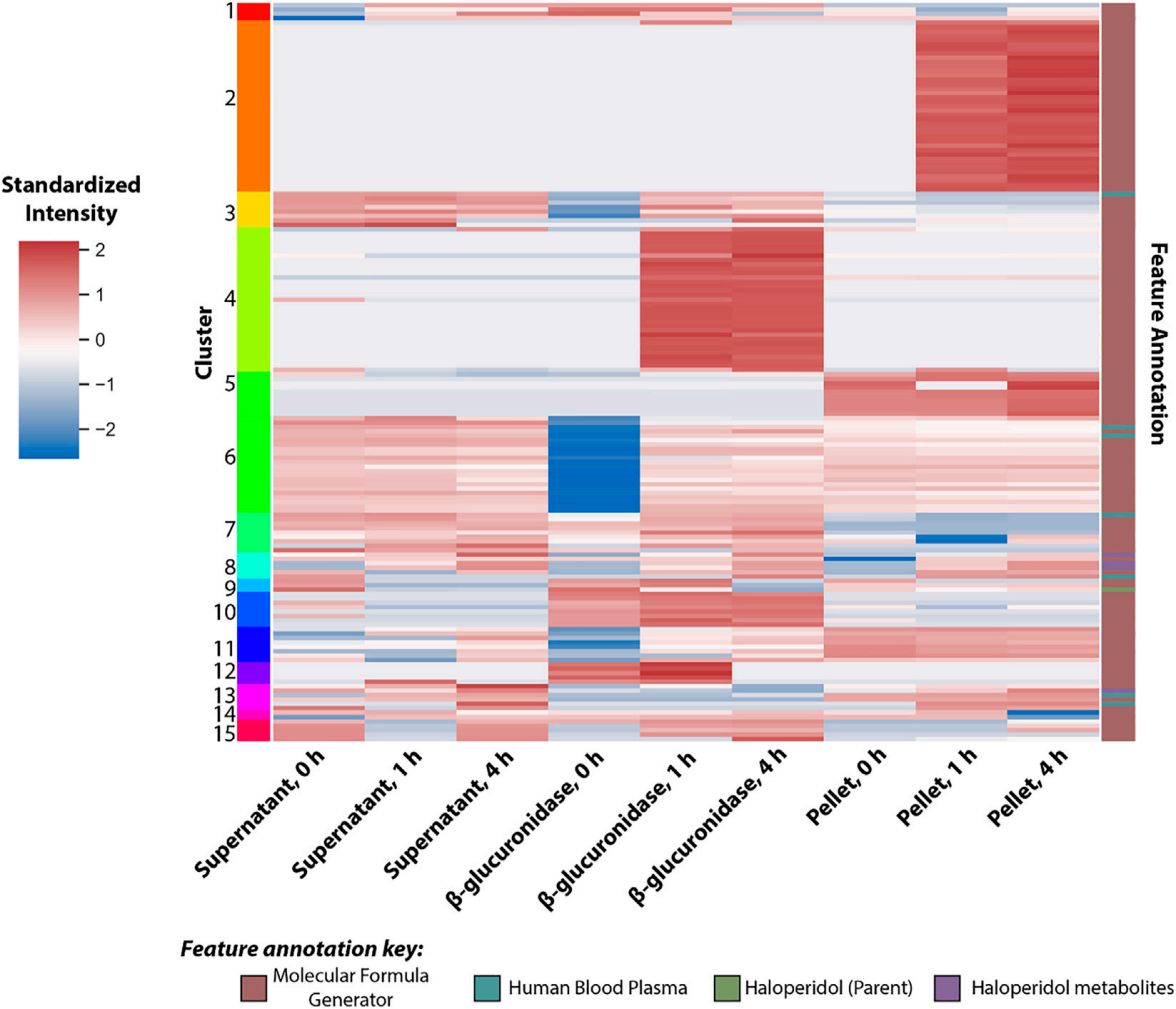
Heat map of features identified in the haloperidol dataset. Each row represents a single feature extracted from the MS^1^ and standardized across all experimental conditions. Features were annotated using either Agilent’s molecular formula generator, a list of metabolites common to human blood plasma, or a list of metabolites related to haloperidol (including the parent structure). The annotation source for each feature is outlined by the right-most column. Features were also clustered into 15 groups (left-most column) based on their distribution across the experimental conditions *via* a K-means clustering algorithm.

Cleaning the data removed duplicate and irreproducible features, as well as features flagged as adducts, and reduced the total of features to 529 when summed across both the positive- and negative-ion mode. The cleaning process removed one feature that had been annotated as a suspected metabolite of haloperidol, leaving ten remaining features annotated as either haloperidol or an associated metabolite. Figure 4 displays a heat map of all the features in which each row represents a single feature extracted from the MS^1^ data. The large number of non-parent or metabolite features in Figure 4 likely stem from changes in the expression of endogenous metabolites between hepatocytes dosed with either the vehicle control or haloperidol. Analysis of endogenous metabolites is outside the scope of this research; however, identification of these features could provide insights to the relationship between xenobiotic exposure and downstream metabolic pathways.

#### 3.3.2 Additional filtering to remove features with aberrant behavior

Metabolites of haloperidol were expected to increase with time. As such, features that failed to show an increase in abundance over the course of the experiment (relative to the 0-h timepoint) were incongruent with the expected behavior of a haloperidol metabolite. Features that were not annotated by the PCDL associated with haloperidol metabolites were removed from the data if they failed to meet two criteria: 1) have a fold-change increase, relative to the 0-h time point, greater than or equal to 1.5 in at least one time point, and 2) be detected in at least two time points across the time series. A total of 170 features met these criteria, with six of these features annotated by haloperidol’s suspect list.

#### 3.3.3 K-means cluster analysis to identify groupings of potential metabolites

**FIGURE 5:**
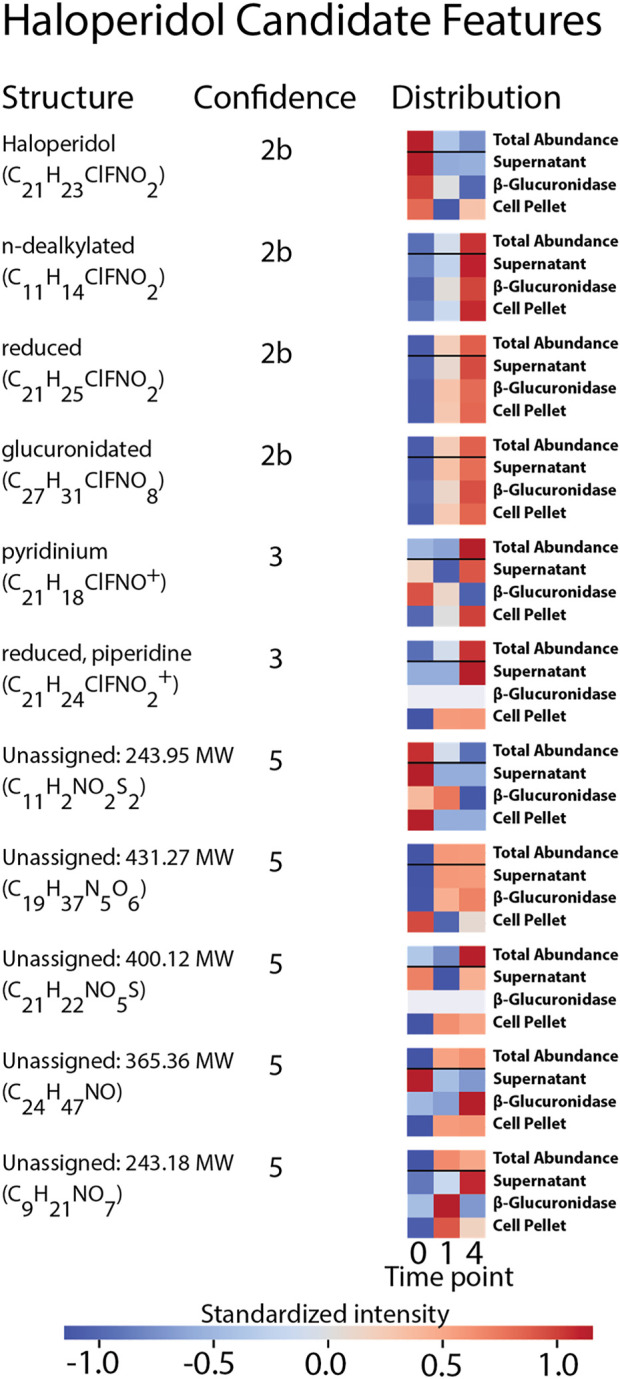
Summary of the structural assignments for each candidate feature selected from the analysis of haloperidol. Confidence in metabolite identification was assigned using three levels of confidence derived from Schymanski et al. (2014).

Features that exhibited similar trends across time and conditions were grouped into 15 clusters using the k-means clustering algorithm. The cluster assignment, annotation source, and standardized signal for each feature are visualized in Figure 5. Features annotated by the Human Blood Plasma PCDL were removed from subsequent identification steps, as these features were annotated as endogenous metabolites and not a transformation product of haloperidol. The feature annotated as haloperidol was present in cluster 9, and five features annotated as haloperidol metabolites were partitioned between clusters 8 and 13. Five additional features annotated with molecular formula and not associated with known or predicted metabolites of haloperidol were also included in clusters 8 and 13. Clusters 2 and 5 contained features that were predominantly measured in the cell pellet, while clusters 4, 10, and 12 contained features that were predominantly measured in the β-glucuronidase-treated samples. Interestingly, features present in clusters 2 and 4 were not detected at t = 0, which suggests these features are metabolic products that developed over the course of the experiment.

#### 3.3.4 Structure assignment using an *in silico* library of MS^2^ spectra and suspect screening list

Eleven features were selected as *candidate features* because they were: 1) annotated by the suspect list and labeled as either haloperidol or a metabolite of haloperidol, or 2) exhibited similar distributions across samples as the annotated metabolites of haloperidol, determined through the cluster analysis. Ten of these features associated with metabolites of haloperidol and one feature was annotated as haloperidol itself. These candidate features were carried forward as features-of-interest for MS^2^ spectral matching.

The precursor masses of the eleven haloperidol candidate features were queried against the *in silico* MS^2^ library to identify matching spectra. Of the eleven queried masses, eight were present as precursor ions in the database and returned 469 candidate structures. Spectral comparisons were performed between each match and candidate structures were ranked according to their quotient scores. The highest ranked match for each feature that corresponded to haloperidol-related compounds within the suspect screening list was assigned as the probable structure for that feature. In all cases of structure assignment, the structures were congruent with the formula annotations assigned by the MS^1^ analysis and each annotation was provided using the suspect screening list. No isomers were present within the list of haloperidol-related substances, so these assignments represent discrete structures. Structural assignments using the MS^2^ library represent the highest degree of confidence provided by this analysis and correspond to a 2b confidence level (probable structure) intended to be consistent with the confidence communication guidelines suggested by Schymanski et al. (2014). Four structures were assigned using this approach: n-dealkylated haloperidol (C_11_H_14_ClNO_2_), reduced haloperidol (C_12_H_23_ClFNO_2_), haloperidol (C_12_H_25_ClFNO_2_), and glucuronidated haloperidol (C_27_H_31_ClFNO_8_).

Having assigned identities with level 2b confidence to 4 of 11 haloperidol candidate features, there remained seven candidate features that were not assigned structures using the *in silico* library. For these seven features no precursor masses were present in the library within the 10 ppm mass error window of the candidate structures. It is important to note that the suspect screening list was updated with structures reported in literature after the generation of the *in silico* spectra library. This discrepancy can lead to structures annotated by the suspect list but not identified *via* MS^2^ spectral matching. Annotated formulae of the features missing from the *in silico* library were cross-referenced against the list of haloperidol-related compounds as a follow-up to identify candidate structures. No structural isomers were present within the suspect list, so these assignments are represented by discrete structures. Reliance on MS^1^ data and the suspect screening-list limits the confidence of these assignments to tentative structures. We determined that these assignments correspond to a confidence level of 3. We justify this assignment on the basis that 1) the masses match to known or predicted metabolites and 2) we have required that the features increase with time. We believe that these two lines of evidence provide orthogonal experimental confirmation of identity. Two of the candidate features had formulae that were captured in the suspect list—pyridinium haloperidol (C_21_H_24_ClFNO_2_^+^), and reduced haloperidol with a piperidine ring (C_21_H_20_ClFNO_2_^+^). The pyridinium metabolite was annotated by the suspect screening list during the initial MS^1^ analysis, whereas the reduced metabolite with piperidine ring was annotated using Agilent’s molecular formula generator.

Having assigned identities with level 3 confidence to two haloperidol candidate features, there remained five candidate features that were not assigned structures using MS^1^ or MS^2^ data. While these features have formula annotations, these annotations were generated by Agilent’s molecular formula generator. It is unclear the degree of confidence that can be assigned these annotations, so these formulae are considered tentative and correspond to a confidence level of 5. A summary of the annotated structures, confidence levels, and sample distributions are provided in Figure 5.

### 3.4 Analysis of additional compounds

**FIGURE 6:**
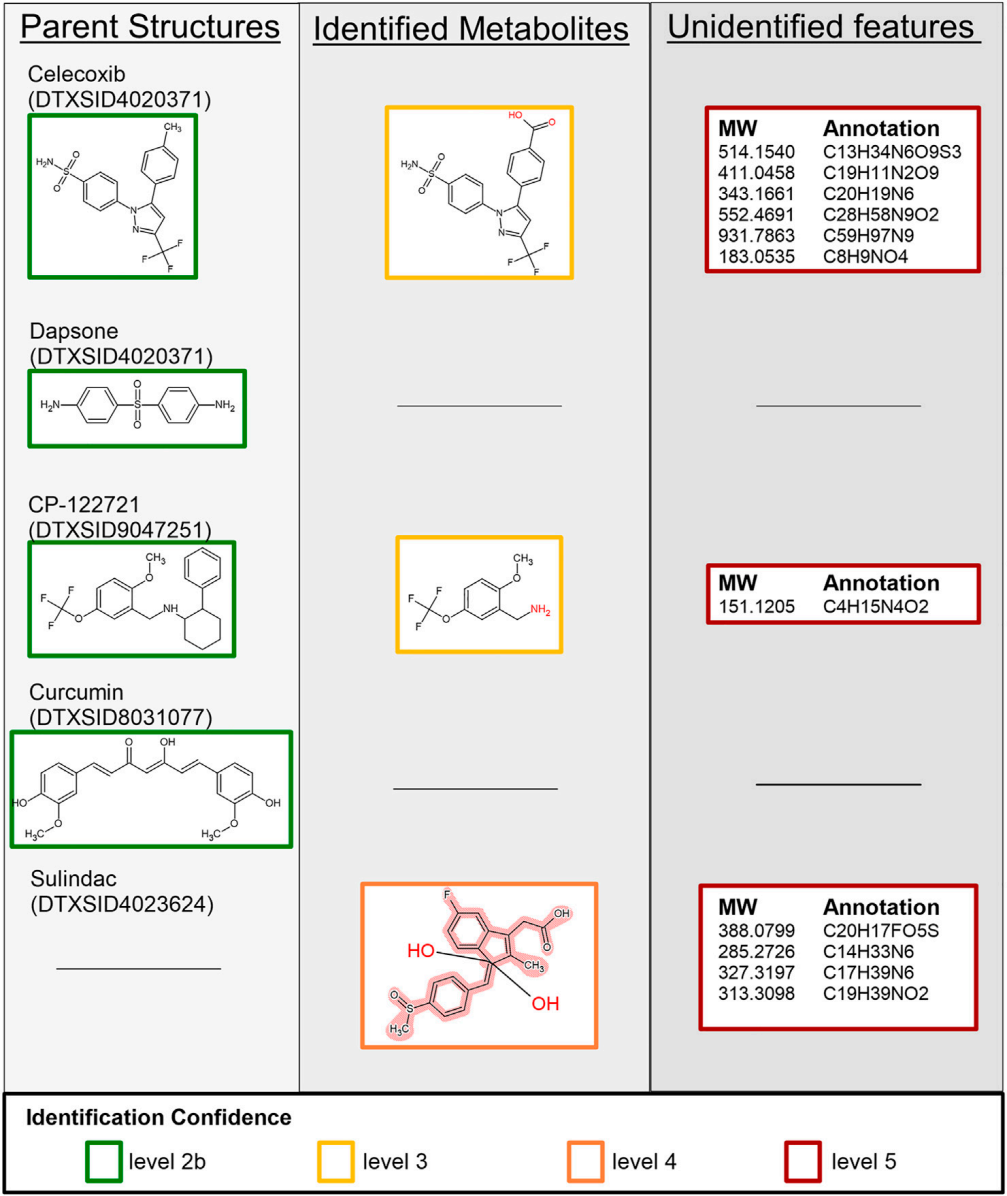
Summary of identified and unidentified features extracted from the MS^1^ and MS^2^ data for celecoxib, dapsone, CP-122721, curcumin, and sulindac. Boxes surrounding individual structures denote the level of confidence in that assignment as described in Section 2.5.4, and the black line indicates that no features were identified to correspond to the respective row and column. Because SSA is not optimized for any one compound, our workflow relied on occurrence of chemical signals in replicate samples and time-correlated changes in signal to filter the signals. In some cases, real chemical detections may have been lost. For example, no features were identified that corresponded to the sulindac parent structure but multiple isomeric sulindac metabolites are represented with a Markush structure. Unidentified features include the molecular weight (MW) and formula annotation.

A subset of additional test chemicals was selected to evaluate the application of the above methodology to other test cases. These compounds were selected from the test chemicals outlined in Figure 3B, with the additional criteria of requiring the parent chemicals to exhibit varying degrees of metabolites to ensure the method is extensible to chemicals undergoing both high and low degrees of metabolism. Celecoxib (DTXSID0022777), curcumin (DTXSID8031077), and CP-122721 (DTXSID9047251) provide representative examples of chemicals that undergo varying degrees of metabolism. Dapsone (DTXSID4020371), and sulindac (DTXSID4023624) show little to no metabolism of the parent chemical. These chemicals were used evaluate whether metabolites can be identified for poorly metabolized parent chemicals. Figure 6 summarizes the structural assignments and confidence levels identified as part of this analysis. Parent structures for four of the starting compounds were identified *via* MS^2^ spectra. Results for each compound are summarized below.

#### 3.4.1 Celecoxib

Nine candidate features were extracted from the MS^1^ dataset with one feature annotated as the parent compound and one feature annotated as a suspected metabolite of celecoxib. Comparisons of the MS^2^ spectra corroborated the identification of the parent compound; however, no identifications were found for the remaining MS^2^ features based on the screening list. The formula of the suspected metabolite was queried against the suspect screening list, and one structure was returned: celecoxib after carboxylation of the toluene moiety. The remaining seven features were unidentified.

#### 3.4.2 Dapsone

One candidate feature was extracted from the MS^1^ dataset, which corresponded to the parent compound. Identification of this feature was corroborated by comparison against the MS^2^ spectra.

#### 3.4.3 CP-122721

Three candidate features were extracted from the MS^1^ dataset, which include a feature annotated as the parent compound and one annotated by the suspect screening list. MS^2^ comparisons confirmed the parent compound, but no suspected metabolites were identified for the suspected metabolite. Only a single candidate structure was returned by the library, and it was not a valid metabolite of CP-122721. Queries against the suspect list were used to assign a tentative structure: N-dealkylation of CP-122721.

#### 3.4.4 Curcumin

Though (Lou et al., 2015) found that curcumin and many of its metabolites are detectable with LCMS, our SSA workflow only confirmed presence of the parent compound. Several curcumin metabolites were included in the suspect list. There were two LCMS signals reported specific to the curcumin sample set with the same molecular formula as the parent. One of the potential metabolites in the suspect screen list had the same molecular formula as the parent so it was plausible one is the parent, and one is a metabolite. There were four signals reported as matching the metabolite list and specific to the curcumin sample set. There was one signal noted as specific to the curcumin sample set but not on the suspect screen list. However, the standardized workflow used here filtered out signals that did not to change with respect to time. Some known curcumin metabolites were present at time zero and the signal (concentration) did not change with time. Other known metabolites were detected at one and 4 h at the same concentration, possibly indicating metabolism faster than 1 h. Only curcumin itself passed the checks of our workflow and was extracted from the MS^1^ dataset. Comparisons of MS^2^ spectra corroborated the identity of this structure.

#### 3.4.5 Sulindac

Five candidate features were extracted from the MS^1^ dataset of sulindac: one feature was annotated as a suspected metabolite *via* sulindac’s PCDL, and the remaining were annotated *via* Agilent’s molecular formula generator. No structures were assigned *via* MS^2^ structural comparisons; however, querying the molecular formula of the suspect metabolite (C_20_H_17_FO_5_S) against the suspect screening list identified 44 potential isomers. Of note, this structure corresponds to the occurrence of two oxidation events, which has not been reported in literature but was predicted by *in silico* tools. The Markush representation is provided in Figure 6.

### 3.5 Comparison of metabolites identified *in vitro* to those reported in literature and predicted by *in silico* tools

**FIGURE 7:**
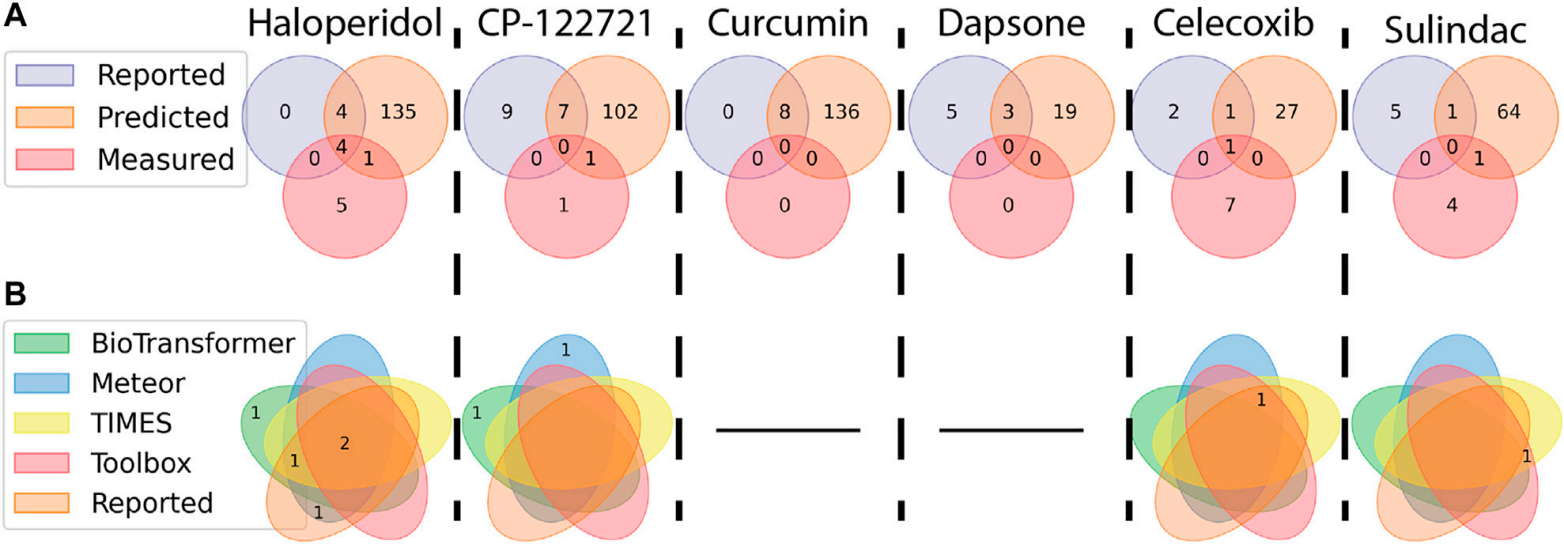
Venn diagrams showing the distribution and similarity of metabolites identified from different sources. Numbers indicate how many metabolites match the conditions of each ellipse. **(A)** Similarity of metabolites are compared between features measured using the NTA method outlined in this work, reported in literature, and predicted by *in silico* tools. **(B)** Metabolites identified as part of the NTA were separated based on the source of the structure (a black line indicates no metabolites were identified *via* the NTA). Taking Haloperidol for example, four metabolites were reported in the literature, predicted *in silico*, and observed. Another four metabolites were reported and predicted, but not observed. One previously unreported metabolite was predicted and observed.

Figure 7A highlights the similarity between the candidate metabolites extracted *via* the SSA method described in this work and metabolites that were reported by literature or predicted by *in silico* tools. In all cases except celecoxib, which had six level 5 features potentially unrelated to the parent (see Figure 6), the total number of metabolites reported in literature were greater than the candidate features identified by this study. This is particularly true of curcumin and dapsone, where no metabolites were identified. These results suggest that the methodology outlined in this work is less sensitive than those summarized in literature. Afterall, the analytical techniques used were not optimized for a single family of chemicals but were kept general to emphasize analysis throughput across a wide range of chemical space. Eight candidate features were assigned structures that overlapped with literature and/or *in silico* predictions, with two structures not previously reported in literature: reduced piperidine haloperidol, and doubly oxidized sulindac. Of the eight assigned structures, there were no instances where metabolites were reported in literature but not predicted by *in silico* tools.

The distribution of assigned metabolites across the *in silico* tools are outlined in Figure 7B. BioTransformer predicted structures for five of the candidate features, with one feature (reduced piperidine haloperidol) predicted solely by BioTransformer. Meteor had the highest number of structural assignments, six, with one assignment (n-dealkylated CP-122721) predicted by no other *in silico* tools. TIMES and the Toolbox predicted five and four structures, respectively, with neither identifying metabolites unique to that software.

## 4 Discussion

The public is potentially exposed to thousands of chemicals that are present in commerce and the environment (National Academies of Sciences, Engineering, and Medicine, 2017). Many of these chemicals have yet to be investigated for health effects. Once inside the body, both the chemicals themselves as well as any xenobiotic metabolites formed have the potential to alter health; but in most cases we do not know what metabolites may occur. Metabolite prediction tools can suggest plausible metabolites that might be formed but these tools have a tendency to predict more metabolites than occur (Boyce et al., 2022). *In vitro* methods allow for the generation of new data on the formation of metabolites but have typically been limited by the need for targeted chemical analysis to quantify the presence of specific metabolites, slowing the discovery of novel metabolites. NTA methods have the potential to enhance *in vitro* metabolism studies but require methods to assign structures/identities to the observed chemical features given that no chemical data may currently exist for novel metabolites. When applied to metabolism studies, NTA has the potential to identify multiple previously unknown metabolites simultaneously, but because we have data gaps in all these steps, we need a discovery-oriented workflow with the goal of identifying potential health risks.

Chemicals in commerce and the environment can require metabolic data regardless of whether those chemicals are amenable to a particular measurement methodology. The LCMS analysis reported here was conducted in parallel with a yet unreported gas-chromotography mass-spectrometry (GCMS) analysis. The 33 chemicals were selected to provided representative coverage of the range of chemicals potentially of interest such that limitations in the methodology were clearly illustrated.

Here we have developed an SSA method to characterize xenobiotic metabolite formation using *in vitro* metabolism assays. The methods outlined in this study are intended to provide a framework for using *in silico* prediction tools to identify xenobiotic metabolites. We investigated 33 test chemicals using LCMS SSA and demonstrated how *in silico* tools can be used to guide the identification of metabolites. We first report on the amenability of the test chemicals to LCMS (that is, we identify which test chemicals were detected by LCMS and whether possible metabolites were also detected in those samples). The NTA features were first limited to a suspect screening list generated with *in silico* metabolism predictors informed by the parent chemical structures. We then implemented a data processing workflow that aligned spectra, annotated features as suspected metabolites, and removed duplicate or highly variable features. The remaining features were clustered on time-varying kinetics, and clusters containing suspected metabolites were selected for spectral comparisons against a library of predicted MS^2^ spectra. We demonstrated the development of this workflow using haloperidol as a case study, then applied this method to five additional test chemicals with varying metabolic behaviors: celecoxib, curcumin, CP-122721, dapsone, and sulindac.

The metabolic assay examined here (suspension of primary human hepatocytes pooled from multiple donors) is just one of many *in vitro* metabolism methods available (Moreau et al., 2022). However, primary hepatocyte suspensions are a well characterized method (Shibata et al., 2002; Gouliarmou et al., 2018; Bowman 2019). Both pharmaceutical companies and regulatory agencies have made extensive use of this assay (Hewitt et al., 2007; Breen et al., 2021). Therefore, the limitations of the assay are well known (Gouliarmou et al., 2018). Limitations include a short period of time for measurement (thus reducing accuracy for compounds with slow metabolism) and the lack of metabolic pathways such as those present in more tissue-like (confluent) conditions. But perhaps the greatest limitation is common to most metabolic assays: the need to develop a chemical analysis method sensitive to each parent chemical and metabolite (Tolonen and Pelkonen, 2015). That is, while the hepatocyte suspension *in vitro* assays themselves are relatively high throughput, the chemical analysis is not. A hope for the application of SSA methods to data generated from these assays was that we might address chemical analysis limitations.

If the chemical analysis method is well matched to detect both the parent chemical as well as most of its metabolites then it works, as shown with haloperidol. However, we have clearly demonstrated that there is no “one-size-fits-all” approach to SSA metabolism studies given the limitations of the particular method (for example, LCMS is unlikely to detect volatile compounds) (Vinayavekhin and Saghatelian, 2010; Dunn et al., 2013). As shown by Figure 3, we observed cases where decrease in the parent was detected, but no metabolites were observed and where metabolites were detected, but not the parent chemical itself–ideally, we need both. Of the 33 starting chemicals, only 12 were measured by LCMS in at least one sample in either positive or negative ion mode, and 17 produced measurable metabolites. The absence of detectable species can stem from several causes: low metabolite concentrations due to poor metabolic activity, limited detection due to poor ionization efficiency or incompatibility with LCMS detection, or poor retention of the species using the outlined chromatographic method. The formation of reactive metabolites will also limit the detection of metabolic products, as these species are often short lived and readily bind to macromolecules. Without the use of reactive metabolite scavengers (Ma and Subramanian, 2006), these molecules will go undetected using the outlined SSA method.

As applications of NTA continue to grow, spectral libraries will need to be expanded to include commonly occuring xenobiotic chemicals and their metabolites. Existing metabolic information tends not to be organized for informatics, that is, made available in a machine readable, database format, (for example: Maurer et al. (2008); Hodgson (2012)). The methodology developed in this study was applied to a selection of parent chemicals that showed a variable range of metabolism. Twenty-five potential metabolites were selected for six of the starting compounds, including twenty not previously reported. Novel or poorly characterized chemicals are often absent from spectral libraries due to a lack of reference spectra. The combination of *in silico* metabolism predictors and SSA facilitated the detection of these potentially novel metabolites. The chemicals identified are intended to provide a first step toward generating the sorts of information needed to make NTA more useful to regulatory science.

The hepatocytes used in the *in vitro* assay were pooled from multiple donors to provide typical levels of expression across the enzymes found in the liver (as verified by the vendor, Thermo Fisher). The *in vitro* system is an imperfect representation of *in vivo* hepatic metabolism: the expression level of metabolizing enzymes in hepatocytes vary depending on individual genetics, chemical exposure history, and location of the hepatocyte within the liver acinus. Beyond the liver, extra-hepatic metabolic pathways exist and would not be captured by the *in vitro* system used. Therefore, it is to be expected that some metabolites formed *in vivo* would not be observed using *in vitro* hepatocytes and other metabolites observed *in vitro* may be overrepresented relative to those formed by mechanisms that are not present in the assay system used here.

The confirmed detection of a predicted metabolite, or tentative identification of a novel metabolite, can inform both the metabolic pathways and biomarkers of effect. Here we have focused on metabolic pathways because we 1) wished to identify groups of chemical analogs that form similar metabolites and 2) made use of an *in vitro* system (primary hepatocytes) that, while biologically relevant, does not cover all relevant toxicological adverse outcome pathways. The identification of chemical surrogates, potentially based on toxicokinetic similarity, helps address large data gaps in public health risk assessment. For example, TK is one of three aspects considered by the Wang et al. (2012) chemical read-across framework for screening-level risk assessment of Superfund chemicals. Metabolic pathways have also been considered when selecting surrogate chemicals for read-across (Wang et al., 2012; Patlewicz et al., 2019), as similar metabolic pathways indicate similar detoxification processes.

Rapid identification of metabolites for xenobiotic substances might be used to help fill data gaps in risk assessment. Our tested chemicals included four test cases with the potential to inform chemical read-across based on metabolites. Unfortunately, most of the chemicals initially intended to serve as chemical analogs to each other were not detected using this methodology. One collection of analogs included 3,5-dinitroanline, 2-nitroaniline, 3-nitroaniline, and 4-nitroaniline. The parent compounds 3,5-dinitroaniline and 4-nitroaniline were detected, but not the 2-nitroaniline and 3-nitroaniline. Metabolite features were detected for 2-nitroaniline and 3-nitroaniline, but manual review of these data could not identify predicted metabolites that corresponded to the observed features and the data were not analyzed further. For the other three cases with expected similar (or divergent) metabolism we ran into methodological challenges both with amenability of compounds to *in vitro* metabolism assay (volatility) and amenability to chemical analysis method (LCMS). No comparisons were possible for the other three sets of chemicals. The parent compound was not detected with LCMS for either methyleugenol or estragole, and no metabolites for estragole. Neither the parent nor any metabolites were detected for 2-nitrotoluene and 4-nitrotoluene. Neither parent nor any metabolites were detected for any of 4-methyl-2-pentanol, 4-methyl-2-pentanone, isopropanol, and acetone. These results highlight the difficulty of measuring small molecules (<100 MW) and molecules without polar moieties *via* LCMS and emphasize the need to include orthogonal methods for detection of a wider chemical space.

GCMS analysis was used as an orthogonal method to analyze features not amenable to LCMS, and further analysis of these data are pending. The preliminary GC data suffered from multiple limitations: First, GC is less sensitive in general than LC (for example–we only inject 1 µL of sample *versus* 20 µL on LC). Second, the GC-amenable chemicals tend to be more volatile. We assume that this volatility makes it more likely that there was greater loss of these compounds throughout the experimental process. For example. The GC-amenable chemicals may have partially outgassed during the *in vitro* metabolism assays. In some cases, there is evidence of evaporation between the performance of the *in vitro* metabolism assays and the analysis of the samples. If one was to repeat this work and target GC-amenable compounds, an increase in the parent concentration plus storage using sample containers designed to minimize volatile losses should be considered (Speen et al., 2022).

Among the compounds that were amenable to LCMS, the *in vitro* metabolism of haloperidol has been well characterized in human liver microsomes (Fang et al., 2001). Six primary metabolic intermediaries or endpoints are known: glucuronidated haloperidol (G-HP), n-dealkylated haloperidol (4-(4-chlorophenyl)-4-hydroxypiperidine, CPHP), haloperidol tetrahydropyridine (HPTP), haloperidol piperidine (HPP+), reduced haloperidol (RHP), and reduced haloperidol piperidine (RHPP+). Four of these chemicals (HPP+, RHPP+, and HTPT) have been shown to have increase affinity towards serotonin transporters as compared to the parent chemical (Wright et al., 1998). One pharmacologically relevant species (HPP+) was identified as part of the SSA, and an intermediate to RHPP+ (HRP) was also identified. Of five additional chemicals analyzed in depth, suspected metabolites were identified for celecoxib, CP-122721, and sulindac.

This study demonstrates that SSA methods can provide a means to rapidly identify and characterize metabolites of xenobiotic chemicals if the SSA methods used are well matched to the parent chemical and its key metabolites. Initial selection of the study chemicals prioritized a wide range of physiochemical properties and did not account for LCMS compatibility. Here we have highlighted the difficulty of applying a generalized analytical method for the characterization of a wide chemical space. However, improvements can be made to refine the approach used here to make it more flexible and applicable for the evaluation of industrial and environmentally relevant chemicals. For example, incorporating additional methods of detection, such as GCMS, would improve the coverage of measurable species. Using amenability models for the chemical analysis method being employed (for example Lowe et al. (2021)), if available, would improve future studies by increasing the fraction of the tested chemicals and their metabolites that are compatible.

## Data Availability

The datasets presented in this study can be found in online repositories. The names of the repository/repositories and accession number(s) can be found below: https://github.com/USEPA/CompTox-ExpoCast-SSAMetabolism.
