# Supplementary figures and images for: Identifying xenobiotic metabolites with in silico prediction tools and LCMS suspect screening analysis
Source: Front Toxicol. 2023 Jan 18;5:1051483. doi: 10.3389/ftox.2023.1051483 (PMC9889941; doi:10.3389/ftox.2023.1051483)

## Slide 1
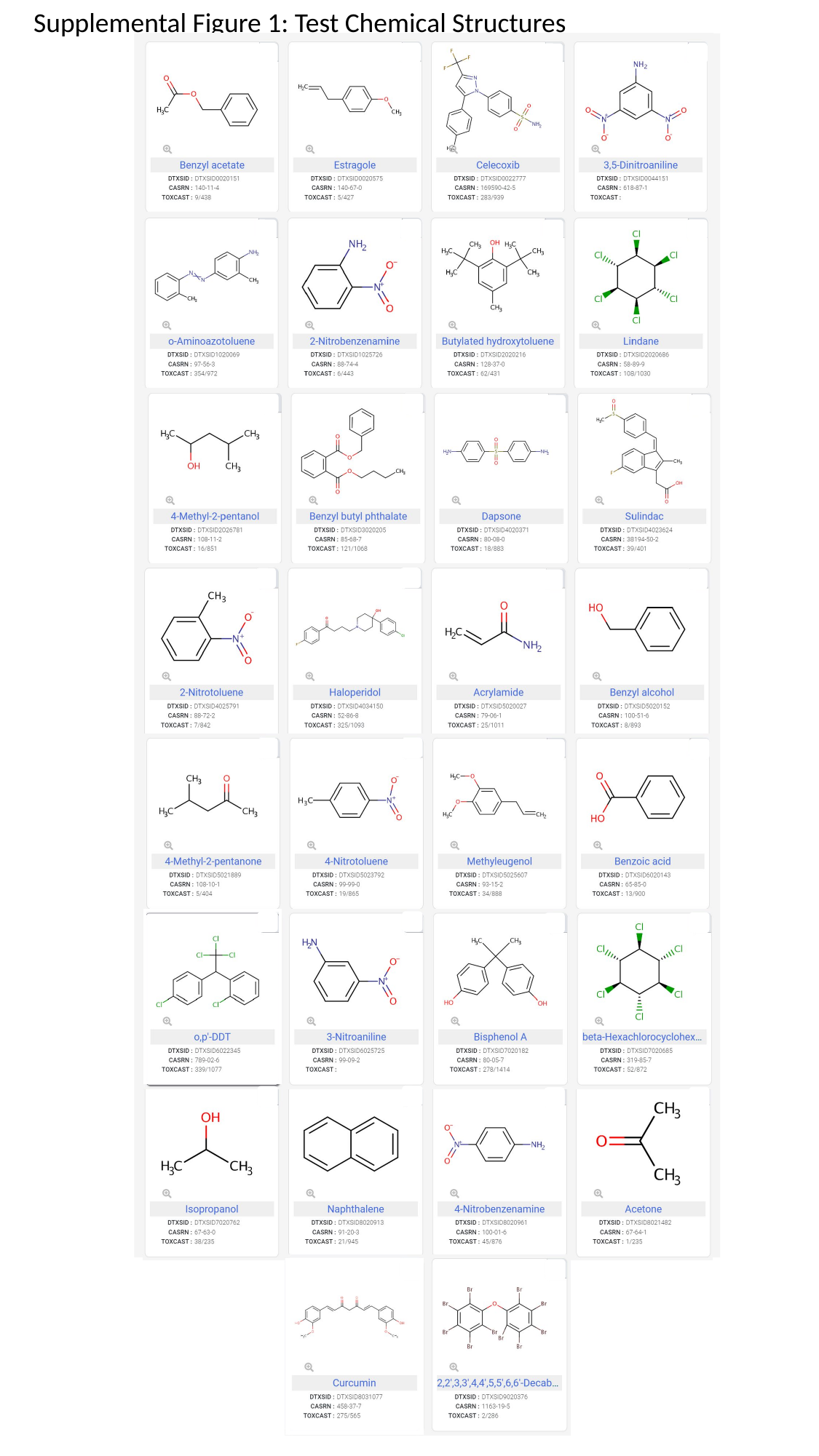

Supplemental Figure 1: Test Chemical Structures

Supplement: Supplementary file 1 [file Presentation1.PPTX]
