# Supplementary material for: Identifying xenobiotic metabolites with in silico prediction tools and LCMS suspect screening analysis
Source: Front Toxicol. 2023 Jan 18;5:1051483. doi: 10.3389/ftox.2023.1051483 (PMC9889941; doi:10.3389/ftox.2023.1051483)
Supplement: Supplementary file 2 [file DataSheet1.docx]

**SUPPLEMENTAL MATERIAL**

**Identifying xenobiotic metabolites with *in silico* prediction tools and LCMS non-targeted analysis**

Table of Contents:

Section S1: Diagram of hepatocyte metabolism assay and sample count

Section S2: Example of Markush representations

Section S3: Settings used for each metabolite prediction software

Section S3: Experimental conditions and instrument parameters for high-resolution mass spectrometry

Section S4: Agilent ProFinder settings for spectral alignment and feature extraction

Section S5: Agilent Mass Profiler Professional (MPP) settings for feature annotation

# Section S1: Diagram of hepatocyte metabolism assay and sample count


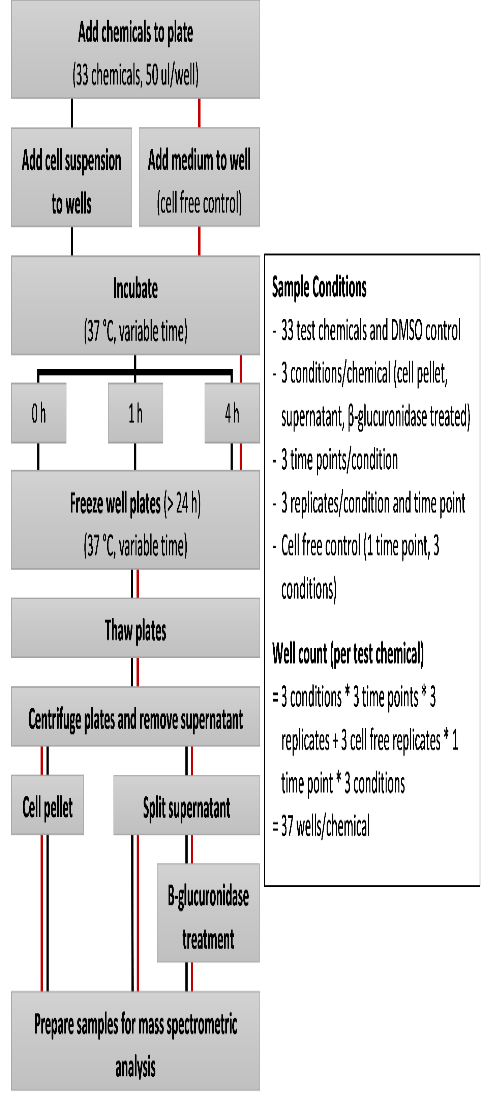

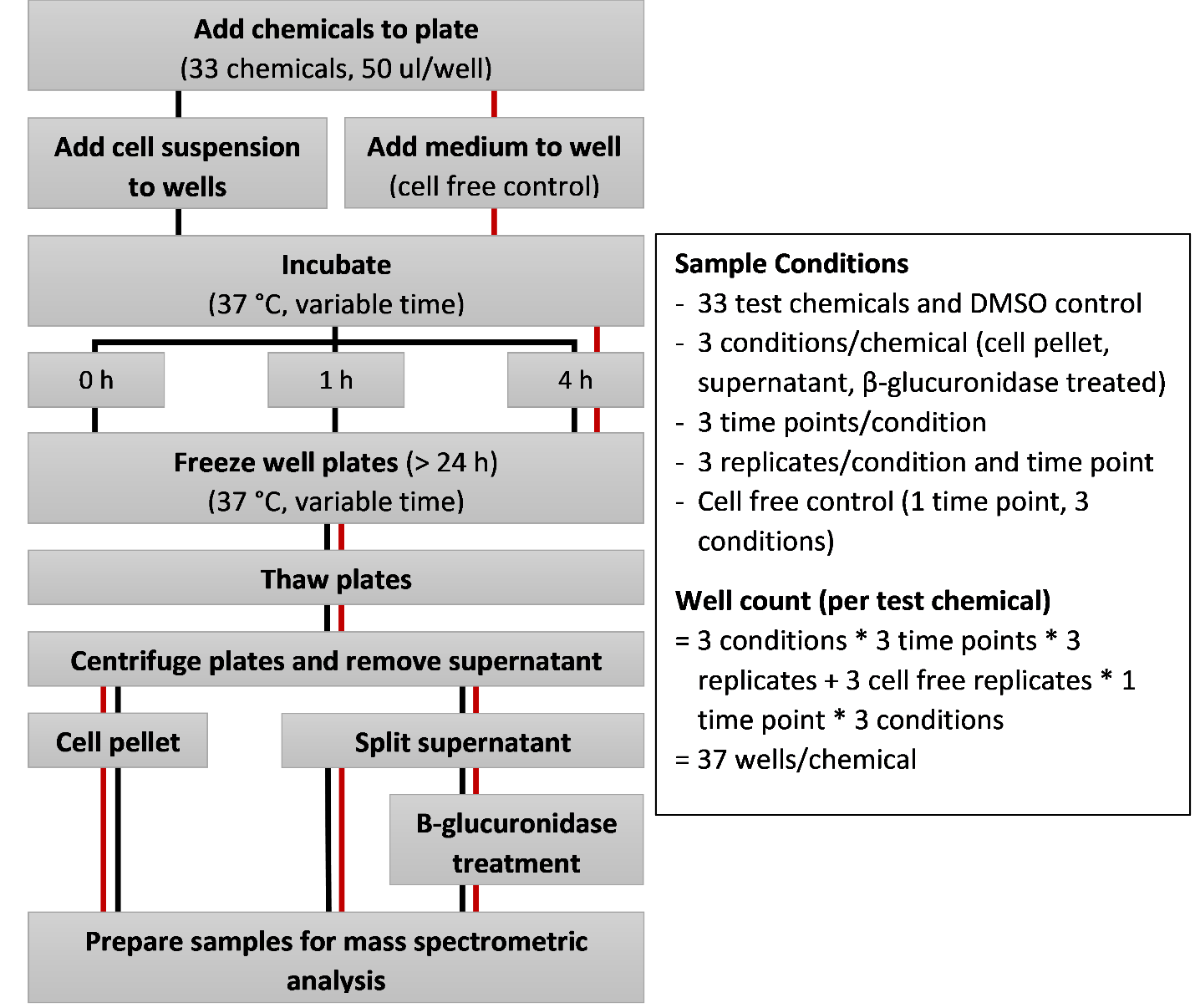

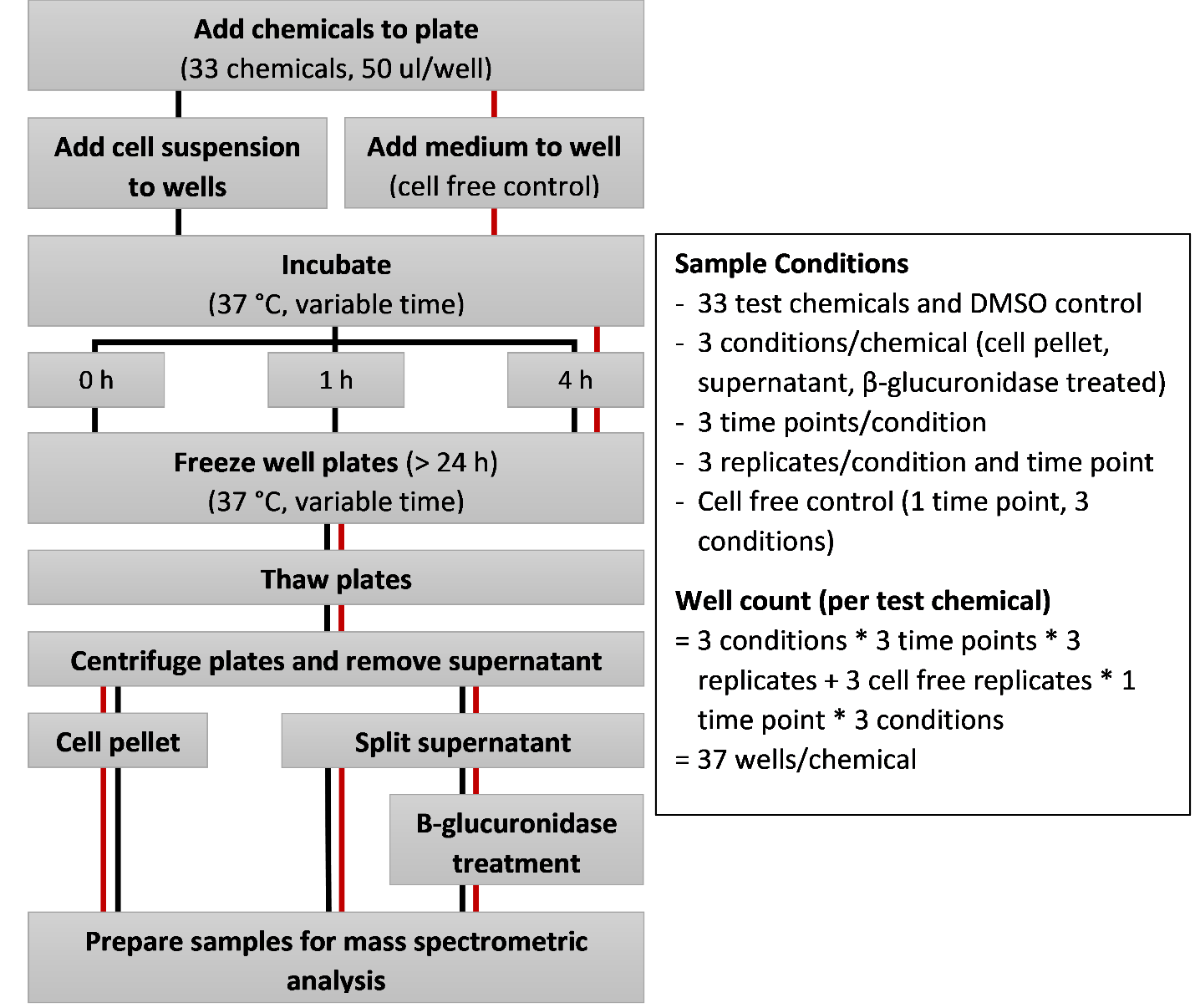


-80

**(>18 h)**

# Section S2: Example of a Markush representation of cresol, which can be enumerated into discrete children structures (*o-*, *m-*, or *p*-cresol)


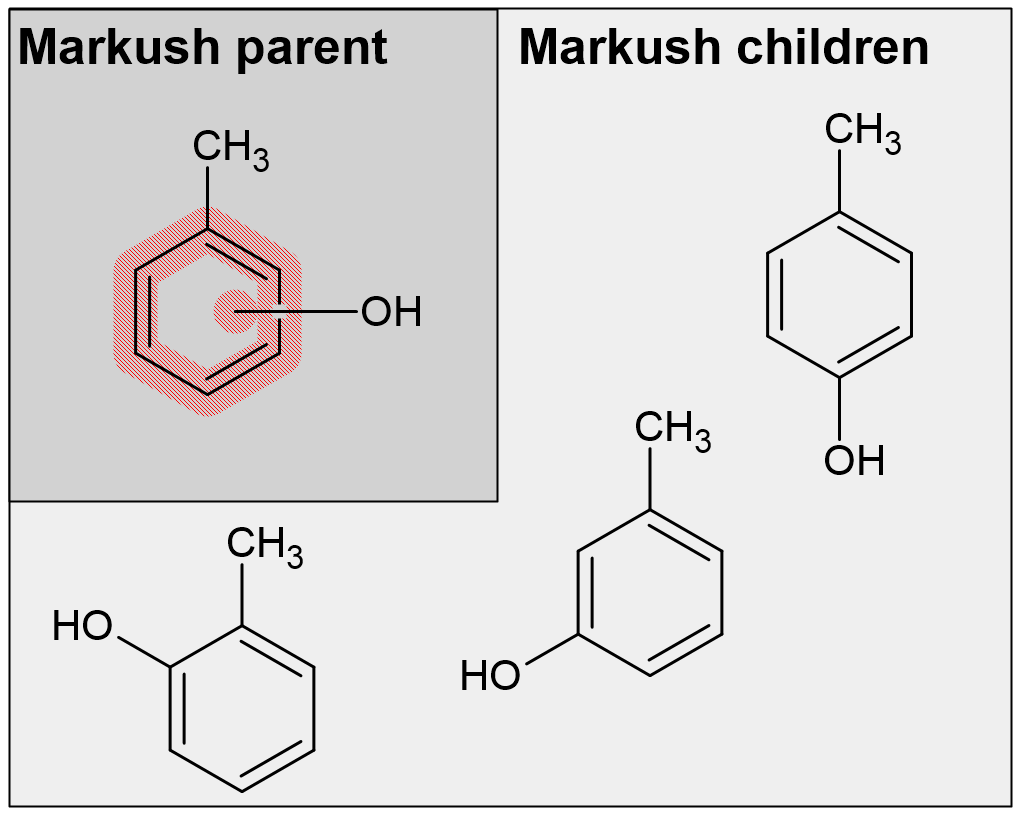


# Section S3: Summary of the metabolite prediction software and their corresponding settings

| **Software** | **Version** | **Settings** | **Modules** |
| --- | --- | --- | --- |
| BioTransformer  (<https://biotransformer.ca>)  (Djoumbou-Feunang et al., 2019) | 2.0 | Cyp450: 2 steps; phase II: 1 step | Cyp450; phase II |
| Meteor BioTransformer (<https://lhasalimited.org>) (Marchant et al., 2008) | 3.1.0 | Default | Mammal |
| QSAR Toolbox (<https://qsartoolbox.org>) (Dimitrov et al., 2016) | 4.5 | Default | Rat (s9), Rat (*in vivo)* |
| TIMES  (<https://oasis-lmc.org>)  (Mekenyan et al., 2004) | 2.30.1.11 | Default | Rat (s9), Rat (*in vivo)* |

# Section S4: Experimental conditions and instrument parameters for high-resolution mass spectrometry analysis of metabolic samples

Metabolite samples were measured using an Agilent 1290 Infinity high performance liquid chromatograph (HPLC) coupled to an Agilent 6540 ultra-high definition (UHD) quadrupole time-of-flight (Q-TOF) mass spectrometer. All samples were measured using positive and negative ion modes. An injection volume of 20 μL was used for each run, and separations were performed using flow rate of 0.3 mL/min and an Agilent Poroshell 120 EC-C18 (2.7 um, 50 x 2.1 mm) column maintained at 50 °C. A solution of 0.1% (v/v) formic acid in water was used as mobile phase A (MPA) and another solution of 0.1% (v/v) formic acid in methanol was used as mobile phase B (MPB). Two internal standards, 3-phenoxy benzoic acid-^13^C_6_ and diisopropylmethylphosphate-D_14,_ were spiked into the sample at a concentration of 100 ppb and used to serve as QAQC checks for negative and positive ion mode, respectively. Mass error and retention time windows calculated for the internal standards were used to establish the corresponding parameters of the Agilent ProFinder and Mass Profiler software. The source conditions and gradient used for this study are summarized in the table below.

| **Source Conditions** | | | **Value** |
| --- | --- | --- | --- |
| Drying gas temperature (°C) | | | 275 |
| Drying gas flow (L/min) | | | 8 |
| Sheath gas temperature (°C) | | | 300 |
| Sheath gas flow (L/min) | | | 11 |
| Vcap (V) | | | 2500 |
| Nozzle voltage (V) | | | 1000 |
| **Gradient** | | | |
| Time (min) | MPA (%) | MPB (%) | Flow rate (mL/min) |
| 0.0 | 98 | 2 | 0.3 |
| 10.0 | 0 | 100 | 0.3 |
| 12.0 | 0 | 100 | 0.3 |
| 12.01 | 98 | 2 | 0.3 |
| 15.0 | 98 | 2 | 0.3 |

Injection sequences were ordered to inject all wells of a single test chemical before advancing to different test chemical, and injections of a single chemical were ordered to inject time points 0, 1, and 4 h for a given condition before advancing to a different condition. Single injections were collected for each well in full scan mode (MS^1^), and the last injection of each triplicate set was injected again to perform targeted fragmentation via data-dependent acquisition (DDA). MS^1^ data were collected using a mass range of 100-1700 m/z and scan rate of 1 spectrum per second, and MS^2^ data were collected using a DDA method with a collision energy of 20 eV. Preferred ion lists were used to guide DDA, where the precursor ions selected for fragmentation corresponded to monoisotopic masses of each test chemical and their respective metabolites ([M+H]^+^ for positive ion mode and [M-H]^-^ for negative ion mode). The preferred ion lists used for each test chemical are provided as a comma-separated value file in the _PCDL folder of the adjoining GitHub repo (). Only a subset of the original 33 chemicals passed the QC, as described in the manuscript.

# Section S5: Agilent Profinder (v. B.08.00) settings for used spectral alignment and feature extraction

| **MFE Extraction Parameters** | |
| --- | --- |
| Peak Height | 10000 (ESI+), 800 (ESI-) |
| Ions (+/-) | Positive mode: +H  Negative mode: -H |
| Isotope Model | ‘Common organic molecules’ |
| Charge State | 1-2 |
| **MFE Compound Filters** | |
| Compound Ion Count | Two or more |
| **Compound Binning and Alignment** | |
| RT Tolerance | +/- 0.00% + 0.04 min |
| Mass Tolerance | +/- 20 ppm + 2.0 mDa |
| **MFE Post-Processing Filters** | |
| Absolute Height |  |
| Score (MFE) | 70.0 |
| Minimum filter matches | 1 file, across all sample files |
| **Find by Ion – Matching Tolerance and Scoring** | |
| Possible M/Z | Symmetric, +/- 35.00 ppm |
| Limit EIC extraction range (Expected retention time) | Yes (+/- 1.50 minutes) |
| Mass score | 100 |
| Isotope abundance score | 60 |
| Isotope spacing score | 50 |
| Retention time score | 0 |
| Expected data variation: MS mass | 2.0 mDa + 5.6 ppm |
| Expected data variation: MS isotope abundance | 7.5% |
| Expected data variation: MS/MS mass | 5.0 mDa + 7.5 ppm |
| Expected data variation: Retention time | 0.115 min |
| **Find by Ion – EIC Peak Integration and Filtering** | |
| Integration algorithm | Agile |
| Smoothing | Gaussian (Function width 9 points) (Gaussian width 5 points) |
| Absolute height EIC filter | 3000 counts |
| **Find by Ion – Spectrum Extraction and Centroiding** | |
| Spectra to include | Average scans > 10% of peak height |
| Exclude if above | 20% of saturation in the m/z ranges used in the chromatogram, never return an empty spectrum |
| Peak spectrum background | none |
| Peak location: Maximum spike width | 2 |
| Peak location: Required valley | 0.7 |
| **Find by Ion – Post-Processing Filters** | |
| Score (Tgt) | 50 |
| Minimum filter matches | 100% of files in at least one sample group (samples grouped as replicates) |

# Section S6: Agilent Mass Profiler Professional (v. 15.1) settings used for feature annotation

| **Data Import Filtering** | |
| --- | --- |
| Minimum absolute abundance | 5000 counts |
| Retention time | Use all available data |
| Mass | Use all available data |
| Minimum number of ions | 2 |
| Charge states | All charge states permitted |
| **Alignment Parameters** | |
| RT Window | 0.0% =/- 0.01 min |
| Mass Window | 0.01 ppm +/- 0.0 mDa |
| **Compound Identification: Identification Workflow** | |
| Compound Identification methods | Identify by – Library / Database search,  Search all Libraries / databases |
| Maximum hits per compound | 10 |
| Identify by – Formula generation | When there are no Library / Database hits |
| **Compound Identification: Database Search Settings** | |
| Mass Tolerance | 10 ppm + 2.0 mDa |
| Positive ions | +H |
| Negative ions | -H, +HCOO |
| Charge state range | 1 |
| DB ion type search mode | Neutrals, Cations, Anions |
| Maximum number of peaks to search when peaks are not specified | 5 |
| Search result: Limit to the best | 10 |
| **Compound Identification: Database Search Settings - Scoring** | |
| Mass score | 100.00 |
| Isotope abundance score | 60.00 |
| Isotope spacing score | 50.00 |
| Retention time score | 100.00 |
| Expected data variation: MS mass | 2.0 mDa +/- 5.6 ppm |
| MS isotope abundance | 7.5% |
| MS/MS mass | 5.0 mDa +/- 7.5 ppm |
| Retention time | 0.115 |
| **Compound Identification: Generate Formula** | |
| Positive ions | +H |
| Negative ions | -H |
| Elements and limits (Element, Minimum, Maximum) | C, 3, 60  H, 0, 120  O, 0, 30  N, 0, 30  S, 0, 5  Cl, 0, 3 |
| Maximum neutral mass for which formulas should be calculated | 1000.00 |
| Minimum overall score per charge carrier | 35.00 |
| Maximum number of hits per charge carrier | 5 |
| Isotope model | Common organic molecules |
| Annotate fragment spectrum peaks with formula | Checked |
| Absolute height | > 10 |
| Relative height | 0.1% largest peak |
| Limit (by height) to the largest | 100 |

# Section S7: **Data cleaning using a custom python script**

Positive- and negative-ion mode peak list data were processed using custom scripts that 1) grouped data based on common conditions (e.g., supernatant, cell pellet, or β-glucuronidase treated), 2) removed duplicate features, 3) calculated summary statistics, 4) removed irreproducible features, 5) flagged potential adducts, and 6) merged results into a single file. The code is stored in GitHub repository (URL) within the ‘Scripts’ -> ‘MS_Analysis’ sub folder. The ‘RunNTA_v1.py’ script coordinates analysis of the peak list input data and imports helper functions from the additional scripts.

1. Grouping Data

The positive- and negative-ion mode peak list data were split into individual files, with each file containing triplicate measurements for each combination of condition, time point, and ionization mode. The DMSO measurements at the matching time point, condition, and ion mode were included with these to sample to serve as a background subtraction at a later step. Each of these files were processed individually for steps 2 -5, then merged back into a single file at step 6.

1. Removing duplicate features

Features were considered duplicates if their measured mass and retention time values were within 10 ppm and 0.05 min, respectively. When duplicate features were found within the data, intensity values for each sample are summed together and the heavier feature is dropped from the feature list.

1. Calculate Statistics

Summary statistics were calculated for the triplicate measurements of each feature in the blank and sample measurements. The median abundance, coefficient of variation (CV), number of hits (i.e., number of replicates the feature was measured in), and background-subtracted median abundance were calculated for the sample features.

1. Removing irreproducible features

Three quality assurance criteria were used to determine whether features were irreproducible: reproducibility across replicates, reproducibility in abundance, and relative abundance compared to the DMSO blank. Features that were only present in a single replicate were not considered to be reproducible across replicates and were removed from the feature list. Similarly, features with a coefficient of variation (CV) greater than 80% for the measured abundance values were considered too variable and were removed from the peak list. Finally, features with an average abundance less than a 3-fold increase over the average blank abundance were not considered insufficiently different from the blank, and were dropped from subsequent analysis.

1. Flagging Adducts

Possible adducts were identifying by calculating mass differences between features that were within a +/- 0.05 min window of one another. Features were flagged as adducts if their mass differences were present in the following table with corresponding ionization mode:

| Mode | Adduct |
| --- | --- |
| Positive | 'Na': 22.989218, 'K': 38.963158, 'NH4': 18.033823, 'H2O': -18.010565, 'CO2': -43.989829 |
| Negative | 'Cl': 34.969402, 'Br': 78.918885, 'HCO2': 44.998201, 'CH3CO2': 59.013851, 'CF3CO2': 112.985586, 'H2O': -18.010565, 'CO2': -43.989829 |

Features that were present as a parent structure to an adduct was labeled as ‘Has_adduct_or_loss’ and features that were identified as an adduct were labeled as ‘Is_adduct_or_loss’. Relationships between adducts and parent structures were stored for each feature by adding an additional data column including all feature IDs of related structures.

1. Merging analysis results

Results from each condition and time point pairing were combined into summary files for each ionization mode. These data were merged using the feature ID’s preserved from the initial peak list input and were used for subsequent statistical analysis for metabolite identification.

# Section S8: Spectra similarity comparisons using a python script

The python scripts used to calculate similarity between experimental data and predicted MS^2^ spectra are stored in a GitHub repository (https://github.com/MBoyce16/XenobioticMetabolismID) within the ‘Scripts’ -> ‘MSMS_Analysis’ sub folder. The ‘run_v24.py’ script serves as the primary module for executing the analysis by importing helper functions and coordinating the main operations: 1) opening and parsing mascot generic files (.mgf) to extract precursor mass and fragmentation spectra for each feature, 2) filtering the precursor mass list to include only those present within the PCDL of the parent chemical, 3) querying a local database containing chemical information and CFMID predictions for each feature mass, 4) calculating similarity between corresponding experimental spectra and CFMID spectra. Similarity is scored using a composite dot-product(Stein and Scott, 1994) with multiple comparisons made between a single experimental spectra and multiple predicted collision energies, at 10, 20 and 40 eV collision energies). The results of the script are exported as two comma-separated files (.csv), one for positive-ion mode and one for negative-ion mode, that include the precursor mass, chemical identifier, and similarity score.

# Section S9: Evaluate of the quality of the MS1 data

Agilent’s Qualitative Analysis software (v. 10.0) was used to automate the extraction and integration of feature peaks in the MS^1^ data. Spectra were first assessed for data quality by generating extracted ion chromatograms (EICs) of internal standards used for positive- and negative-ion mode. The EICs were generated by searching for monoisotopic masses ± 10 ppm of the [M+H]^+^ or [M-H]^-^ ions. The distribution of retention time, peak area, and mass error values of the internal standards were plotted for each test chemical (figure S2) and are provided on GitHub: https://github.com/MBoyce16/XenobioticMetabolismID/blob/main/QAQC%20Data/QAQC_data.xlsx. The range of mass error and retention times values measured for each internal standard were used to set the feature alignment ranges for subsequent feature selection.

# References

Dimitrov, S., Diderich, R., Sobanski, T., Pavlov, T., Chankov, G., Chapkanov, A., Karakolev, Y., Temelkov, S., Vasilev, R., and Gerova, K. (2016). QSAR Toolbox–workflow and major functionalities. *SAR and QSAR in Environmental Research* 27**,** 203-219.

Djoumbou-Feunang, Y., Fiamoncini, J., Gil-De-La-Fuente, A., Greiner, R., Manach, C., and Wishart, D.S. (2019). BioTransformer: a comprehensive computational tool for small molecule metabolism prediction and metabolite identification. *Journal of cheminformatics* 11**,** 1-25.

Marchant, C.A., Briggs, K.A., and Long, A. (2008). In silico tools for sharing data and knowledge on toxicity and metabolism: Derek for windows, meteor, and vitic. *Toxicology mechanisms and methods* 18**,** 177-187.

Mekenyan, O.G., Dimitrov, S.D., Pavlov, T.S., and Veith, G.D. (2004). A systematic approach to simulating metabolism in computational toxicology. I. The TIMES heuristic modelling framework. *Current pharmaceutical design* 10**,** 1273-1293.

Stein, S.E., and Scott, D.R. (1994). Optimizing and Testing of MAss Spectral Library Search Algorithms for Compound Identification. *J. Am. Soc. Mass Spectrom.* 5**,** 859-866.
